# Influence of genetic biomarkers on cardiac diseases in childhood cancer survivors: a systematic review

**DOI:** 10.1038/s41397-025-00369-y

**Published:** 2025-05-24

**Authors:** Naïla Aba, Claire Ducos, Eric Morel, Chiraz El Fayech, Brice Fresneau, Florent de Vathaire, Gwénaël Le Teuff, Nadia Haddy

**Affiliations:** 1 Université Paris-Saclay, UVSQ, Inserm, CESP, Villejuif, France; 2 CESP, INSERM U 1018, Cancer and Radiation team, Gustave Roussy, Villejuif, France; 3 Inserm, U769, Univ. Paris-Sud 11, IFR141, Labex Lermit, Châtenay-Malabry, France; 4 Department of Childhood and Adolescent Oncology, Gustave Roussy, Villejuif, France; 5 Department of Research, Gustave Roussy, Villejuif, France; 6 Bureau de Biostatistique et d’Epidémiologie, Gustave Roussy, Villejuif, France; 7 Université Paris-Saclay, CESP, INSERM U1018 Oncostat, labeled Ligue Contre le Cancer, Villejuif, France

**Keywords:** Risk factors, Genetics research, Public health, Disease prevention

## Abstract

Childhood cancer survivors (CCS) often suffer from cardiac disease (CD) after treatment that included anthracycline and radiotherapy involving the heart. However, the variability in CD occurrence cannot be explained solely by these treatments, suggesting the existence of genetic predisposition. We conducted a systematic review searching on Medline-PubMed and Scopus, to identify studies reporting associations between genetic factors and CD in CCS. We included studies published up to 11 April 2023, with no lower limit, and assessed the quality of genetic associations by the Q-genie tool. As a result, 20 studies were included (15 case-control and five cohorts), revealing several genes and variants associated with cardiomyopathy, among which, *SLC28A3*-rs7853758, *RARG*-rs2229774, *P2RX7-*rs208294 and *P2RX7-*rs3751143 variants gave the most consistent findings. This review highlights the necessity to establish a set of clinically useful genes and variants to identify patients most at risk of developing cardiomyopathy, and to implement monitoring and prevention strategies.

## Introduction

Five-year survival after childhood cancer treatment has improved significantly over the past five decades [1], with a rate exceeding 80% in the 2000s [2]. Unfortunately, these therapeutic advances are associated with multiple secondary diseases at a relatively young age in childhood cancer survivors (CCS) [2, 3]. Cardiac diseases (CD) are the major non-malignant cause of death in this population [2–7], and CD risk increases in CCS treated with anthracyclines [8] and radiotherapy (RT) involving the heart [4, 8, 9]. These CD can manifest as cardiomyopathy and heart failure [10–12], but also as coronary artery disease, valvular disease, arrhythmias and conduction disorders, and pericardial disease [4, 10].

The mechanisms underlying radiation and anthracycline treatment-induced cardiotoxicities are multifactorial and include mitochondrial dysfunction, oxidative stress, apoptosis, and dysregulation of autophagy [13, 14]. Several other risk factors associated with CD have been identified, such as a younger age of exposure to cancer treatment, female sex, comorbidities, and African ethnicity [15]. However, the ability of models including these factors to predict patients at risk of cardiotoxicity remains limited [6, 16]. Indeed, variability in the occurrence of cardiomyopathy has been observed in similarly treated CCS, suggesting the existence of inter-individual predispositions [3]. Some studies have investigated the role of genetic factors mainly on cardiomyopathy occurrence after treatment of childhood cancer with anthracyclines and/or RT involving the heart. These studies used different sequencing approaches, such as candidate genes, focusing on one or several specific variants, genome-wide association studies (GWAS), whole-exome sequencing (WES), and whole-genome sequencing (WGS), and aim to identify genes and variants associated with cancer treatment-related cardiomyopathy.

Identifying genetic biomarkers is essential to improve the prediction of CD risk after childhood cancer treatment. This would enable more accurate classification of CCS into risk categories based on their individual susceptibility to CD. Since not all CCS exposed to the same treatments develop CD [6], understanding genetic differences can refine risk stratification, allowing targeted cardiac monitoring for high-risk patients. Furthermore, early identification of at-risk individuals at the time of cancer diagnosis could guide the adaptation of therapies to prevent cardiotoxicity and improve long-term outcome [3, 17]. We performed a systematic review to summarize the published findings on the genetic factors associated with CD in CCS and report the potential interactions of genes/variants with cancer treatments.

## Materials and methods

### Search strategy and selection criteria

This systematic review was registered on PROSPERO CRD N°2023388689. Literature research was performed to identify studies published before April 11, 2023, reporting genetic associations between genes/variants and CD in CCS. The research was and further updated on August 29, 2024. In order to minimize the heterogeneity in the selected studies, we used the PICOS (Population, Intervention, Comparison, Outcome, Study design) framework, and defined clear criteria of inclusion: Population – patients treated for childhood cancer before 21 years; Intervention – patients treated with chemotherapy and/or radiotherapy; Comparator – not applicable for this review; Outcomes – papers that included genetic data involved in CD risk in patients treated for childhood cancer; Study – case-control and cohort studies (Appendix Table A).

The first part of the search strategy involved searching in the PubMed/MEDLINE and Scopus electronic databases. The second part is a manual search from references cited in the eligible studies identified in the first part to include omitted studies. Details of the complete search strategy are provided in Appendix A.

Two independent reviewers (NA and CD) screened the titles and abstracts to determine whether a full-text review should be performed based on inclusion criteria (Appendix B) to minimize excessive diversity but still capture essential variability. One investigator (NA) screened full-text articles to assess compliance with the selection criteria. If there was any uncertainty about the inclusion of an article, it was reviewed by the second investigator (CD), and the decision was made by mutual agreement.

### Data collection, quality control, and risk bias evaluation

Information from eligible studies was recorded from the main text and supplementary material using a data extraction form (Appendix Table C) comprising 14 items relating to study design, sequencing method, sample size, country of origin and/or ethnicity, cancer type, methods and duration of follow-up, cancer treatment information, cancer treatment or cancer diagnosis area, age at cancer diagnosis, cardiac outcome definition, case and controls definition, gene selection method, statistical analysis, selected genes and/or variants and their association risks with CD.

Study quality was evaluated using the Q-Genie tool which was specially developed to assess the global quality of genetic association studies, and was preferred to other quality assessment tools such as the Newcastle-Ottawa Scale and the Cochrane Risk of Bias tool because these latter may not adequately capture the methodological complexity specific to genetic research [18]. The Q-genie tool consists of a total of 11 characteristics rated from “Poor” (score = 1) to “Excellent” (score = 7). The quality score is the sum of these ratings, with scores ≤35 indicating poor quality study, >35 and ≤45 moderate quality study, and >45 good quality study [18]. A study with a poor quality is excluded from the systematic review.

Data reporting followed Preferred Reporting Items for Systematic Reviews and Meta-Analysis (PRISMA) [19] guidelines.

## Results

### Study, patient data description

**Fig. 1:**
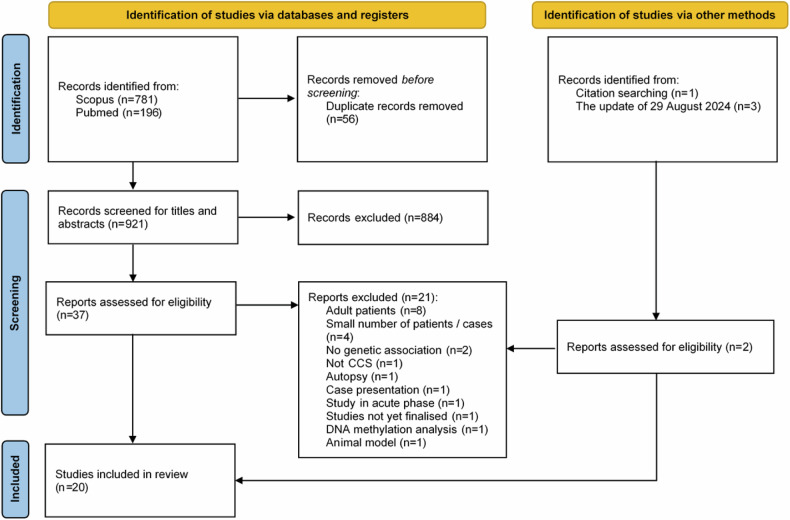
PRISMA flow diagram outlining search strategy and the final included and excluded studies.

The search strategy identified 982 studies published between 1975 and March 2023, of which 56 were duplicates. Of the remaining 922 studies, 884 records did not meet inclusion criteria after screening titles and abstracts (main exclusion criteria: study out of scope, no genetic findings), leading to 39 studies. Finally, 20 studies were selected on the full-text screening [20–39] (Appendix Table D1) and the 21 other studies were excluded mainly because they were related to adult patients (*n* = 8) or a small number of patients (*n* = 4) (Appendix Table D2). The identification and screening process results are detailed in a PRISMA flow diagram (Fig. 1). The 20 included studies were classified as moderate (*n* = 4), and good quality (*n* = 16) according to the Q-Genie tool (Appendix Table E).

Table 1 reports the main characteristics of the 20 included studies published between 2008 and 2023. These studies involved patients diagnosed before the age of 21 with various types of malignant tumors: acute lymphocytic leukemia (ALL, *n* = 4) [33–35, 37], ALL and osteosarcoma (*n* = 1) [31], and unspecified tumors (*n* = 15) [20–30, 32, 36, 38, 39]. All studies reported administration of anthracyclines to patients, and 15 studies (75%) reported treatment with RT involving the heart. For the former, cumulative doses of anthracyclines were specified for each patient. For the latter, the exposure was mainly categorized as “received thoracic radiation: yes/no” [20–23, 25, 27, 28, 30, 32, 38, 39], while five studies considered the heart radiation doses in the analyses, using metrics such as cumulative dose [24, 26] and mean dose [29, 36]. Five studies reported the use of Dexrazoxane, a medication used in the management and treatment of anthracycline-induced cardiotoxicity [30, 33–35, 37] (Details in Supplementary Table 1).

**Table 1:** Characteristics of the 20 included studies.

Study	Country/ ethnicity	Tumor type	Age at cancer diagnosis/ treatment (years)	Follow-up (years)	Treatments (anthracyclines/radiotherapy)	Sequencing method	Study design	Cohort size (case vs. controls)
Sági et al. BMC Cancer, [31]	Hungary	Acute Lymphoblastic Leukaemia and osteosarcoma	Median (range) ALL: 5.2 (0–18) OSC: 13.2 (5 –18)	NA	Anthracyclines (all patients)	Candidate Gene	Case-control study	661 (20 vs. 641)
Blanco et al. JCO, [25]	North America	Any cancer type	Median (range) Cases: 7.3 (0–20.7) Controls: 7.6 (0–21.1)	Median (range) 9.7 (0.1–40.3)	Anthracyclines and/ or radiotherapy	Candidate Gene	Matched case-control study	487 (170 vs. 317)
Visscher et al. Pediatr Blood Cancer, [21]	Netherlands and Canada	Any cancer type	Median (range) Cases: 9.9 (0.5–17.0) Controls: 8.16 (0.5–17.7)	Median (range) 13.8 (0.4–31.6)	Anthracyclines +/− radiotherapy	Candidate Gene	Case-control study	344 (78 vs. 266)
Singh et al. Cancer, [28]	Asian, Black, Hispanic, White	Any cancer type	Median (range) Cases: 7.8 (3.8–11.5) Controls: 9.6 (3.3–14.8)	Median (range) 9.3 (1.3–17.2)	Anthracyclines +/− or radiotherapy	Candidate Gene	Matched case-control study	167 (75 vs. 92)
Blanco et al. Cancer. [24]	North America: Black, White, Others	Any cancer type	Mean (SD) Cases: 10.3 (6.5) Controls: 9.1 (5.8)	≥ 5 years after diagnosis	Anthracyclines +/− radiotherapy	Candidate Gene	Nested matched case-control study	140 (29 vs. 111)
Hildebrandt et al. Nature, [32]	White, Hispanic, Black and other	Any cancer type	Mean (SD) Cases: 9.2 (4.7) Controls: 9.3 (5.7)	Mean (SD) Cases: 21.2(11.2) Controls: 15.7(7.6)	Anthracyclines +/− radiotherapy	Candidate Gene	Matched case-control study	108 (46 vs. 62)
Aminkeng et al. Nat Genet. [23]	Canada: European and non-European ancestry	Any cancer type	Median (IQR) Cases: 8.5 (4 –14) Controls: 5 (2–10)	NA	Anthracyclines +/− radiotherapy	GWAS	Case-control study	376 (54 vs. 322)
Visscher et al. Pharmacogenomics, [22]	Netherlands and Canada	Any cancer type	Median (range) Cases: 9.9 (0.5–17.0) Controls: 4.9 (0.1–17.7)	Median (range) 13.8 (0.4–31.6)	Anthracyclines +/− radiotherapy	GWAS	Case-control study	335 (78 vs. 257)
Wang et al. JCO, [27]	Non-Hispanic whites	Any cancer type	Median (range) Cases: 7.5 (0–20) Controls: 8.3 (5–8)	Median (range) 11.7 (0.1–41)	Anthracyclines and/ or radiotherapy	GWAS	Matched case-control study	331 (112 vs. 219)
Wang et al. JCO, [26]	Non-Hispanic whites	Any cancer type	Median (range) Cases: 6.9 (0–20.2) Controls: 6.3 (0–20.6)	Median (range) 10.87 (0.1–41.0)	Anthracyclines and/ or radiotherapy	GWAS	Matched case-control study	287 (93 vs. 194)
Wang et al. JCO, [38]	North America: Non-Hispanic white,	Any cancer type	Median (range) Cases: 7 (0–20) Controls: 11 (0–20)	Median (range) Cases: 22 (0–39) Controls: 31 (14–45)	Anthracyclines +/− radiotherapy	GWAS	Case-control study	1866 (126–1740)
Visscher et al. JCO, [20]	Canada: European and non-European ancestry	Any cancer type	Median (range) Cases: 6.1 (0.04–17.6) Controls: 3.8 (0.05–16.9)	Median (range) 8.2 (0.1–18.6)	Anthracyclines +/− radiotherapy	GWAS	Case-control study	156 (38 vs. 118)
Chaix et al. JACC : Cardiooncology, [30]	North America	Any cancer type	Median (IQR) Cases: 4.0 (2.0–7.0) Controls: 6.0 (2.0–10.0)	Median (IQR) Cases: 9.0 (6.0–12.3) Controls: 8.5 (5.0–12.3)	Anthracyclines +/− radiotherapy	WES	Nested case-control study	289 (183 vs. 106)
Sharafeldin et al. JACC: Cardiooncology, [39]	USA	Any cancer type	Median (range) 7 (0–21)	Median (IQR) Mild cases: 8.58 (0.3–35.1) Severe cases: 7.46 (0.22–30.3) Controls: 14.44 (1.9–38.9)	Anthracyclines and/or radiotherapy	WES	Matched case-control study	278 (129 vs. 149)
Krajinovic et al. The Pharmacogenomics Journal, [33]	Canada: Caucasian	Acute Lymphoblastic Leukemia	Median (range) 5.0 (1.0–18.0)	Median (range) 8.00 (1.00–18)	Anthracyclines +/− radiotherapy	Candidate Gene	Cohort study	251
Semsei A., Cell Biol. Int, [34]	Hungary	Acute Lymphoblastic Leukemia	Mean (range) 5.7 (1.2–18.0)	Median (range) 6.3 (2.4–13.7)	Anthracyclines (all patients)	Candidate Gene	Cohort study	235
Lipshultz S., Cancer, [35]	Missing	High risk Acute Lymphoblastic Leukemia	59%: <10 years old 41%: ≥10 years old	Median (range) 7.0 (1.1–17.1)	Anthracyclines	Candidate Gene	Cohort study	184
Petrykey et al. Pharmacogenomics, [37]	Canada: European ancestry	Acute Lymphoblastic Leukemia	Median (range) 4 (0–18)	Median (range) 13 (3–24)	Anthracyclines (all patients)	WES	Cohort study	233
Sapkota et al. JNCI J Natl Cancer Inst, [36]	USA: European ancestry; African ancestry;	Any cancer type	Median (range) Cases: 10.5 (0.0–20.5) Controls: 6.9 (0.0–23.6)	NA	Anthracyclines and/ or radiotherapy	WGS	Cohort study	1870 (227–1643)
Sapkota et al. AACR, [29]	USA: African ancestry	Any cancer type	Median (range) 8.8 (0.1–19.7)	NA	Anthracyclines and/ or radiotherapy	WGS	Cohort study	246 (40 vs. 206)

*SD* standard deviation, *IQR* interquartile range, *NA* not available.

### Genetic sequencing

Genetic sequencing was performed on various biological specimens taken from peripheral blood (*n* = 9) [27, 29–32, 34–37], buccal cells/saliva (*n* = 1) [24], and blood or buccal cells/saliva (*n* = 7) [21, 22, 25–27, 38, 39]. Three studies did not specify the biological sample used for the genotyping [20, 23, 33]. Different genotyping methods were used: candidate gene analyses (*n* = 9) [21, 24, 25, 28, 31–35], GWAS (*n* = 6) [20, 22, 23, 26, 27, 38], WES (*n* = 3) [30, 37, 39], and WGS (*n* = 2) [29, 36] (Details in Supplementary Table 2).

### Cardiac disease outcomes

Although no restriction in the search strategy was performed about the type of CD outcome, all the included studies investigated cardiomyopathy defined either by diagnosis or by monitoring echocardiographic parameters. Cardiomyopathy diagnosis was designated by the authors using different denominations: cardiomyopathy (*n* = 5), cancer treatments induced cardiomyopathy (*n* = 1), anthracycline-induced cardiotoxicity (*n* = 4), or clinical cardiotoxicity (*n* = 4). Some authors used congestive heart failure (CHF) as the outcome (*n* = 1), which is a consequence of cardiomyopathy. The monitoring of echocardiographic parameters revealing a decrease in LVEF and/ or in left ventricular fractional shortening (LVFS) (*n* = 5) constituted another outcome [29, 33–35, 37]. For simplicity, the wording cardiomyopathy is used in the rest of the paper to cover the different denominations reported above (Details of outcomes per study are given in Supplementary Table 3).

### Design and statistical methods

Among the 20 selected studies, 15 were case-control studies [20–28, 30–32, 38, 39], including seven matched studies, with case numbers ranging from 38–277. The remaining five were cohort studies [29, 33–37], with patient numbers ranging from 184–1870. Sixty-five percent (*n* = 13) of the studies replicated their findings using independent data from an external cohort (*n* = 5) [20–23, 39] or from patients of the overall cohort not included in the discovery set [26, 27, 29, 30, 33, 36–38]. Only eight studies investigated the effect of variants according to cumulative anthracycline doses. Multiple testing corrections were applied in all GWAS, WES and WGS studies, as well as in four of the nine candidate gene studies. More details on the statistical methods used are reported in Supplementary Table 4.

### Summary of genetic findings

Regarding the heterogeneity in outcomes and sequencing methods in the 20 included studies, we reported the results of genetic associations according to study design (case-control and cohort), which is strongly related to study outcome and the sequencing methods. Table 2 reports the main results, and Table 3 summarizes the effect of variants according to cumulative doses of anthracyclines.

**Table 2:** Main genetic associations’ findings in 19 of the 20 included studies.

Study	Tumor type and Cohort size (case vs. controls)	Sequencing method	Outcome	Gene/ region	Variant	Effect allele	OR (95% CI)	p-values	Correction for multiple testing
Sági et al. BMC Cancer, [31]	ALL and osteosarcoma *N* = 661 (20 vs. 641)	Candidate gene (70 SNP in 26 genes)	Cardiotoxicity	CYP3A5	rs4646450	TT	7.25 (1.83–28.78)	4.81 × 10^−3^	FDR control
				SLC28A3	rs7853758	AA	9.84 (1.73–56.02)	1.00 × 10^−2^	
			LVFS decrease 5 to 10 years after diagnosis	ABCC2	rs3740066	GG/ GA/ AA	39.1 vs. 40.6 vs. 42.4^b^	7.11 × 10^−4^	
				NQO1	rs1043470	TT/ CC+CT	38.5 vs. 40.6^b^	5.82 × 10^−3^	
				SLC22A6	rs6591722	AA/ TA+TT	37.5 vs. 40.6	1.71 × 10^−3^	
Blanco et al. JCO, [25]	Any cancer type *N* = 487 (170 vs. 317)	Candidate gene (2 SNP in2 genes)	Cardiomyopathy	CBR3	rs1056892	GG	1.79 (1.08–2.96)	0.02	No control
				CBR1	rs9024	GG	0.81 (0.45–1.47)	0.49	
				Combine CBR3 and CBR1	Combine: rs1056892/ rs9024	GG+GG	1.53 (0.93–2.51)	0.09	
Visscher et al. Pediatr Blood Cancer, [21]	Any cancer type *N* = 344 (78 vs. 266)	Candidate gene (23 SNP)	ACT	SLC28A3	rs7853758	A	0.36 (0.22–0.60)	1.6 × 10^−5^	No control
				SLC28A3	rs885004	A	0.34 (0.20–0.60)	3.0 × 10^−5^	
Singh et al. Cancer, [28]	Any cancer type *N* = 167 (75 vs. 92)	Gene candidate (1 gene)	Cardiomyopathy	GSTM1	Null/ Positive		2.52 (1.2–5.2)	0.01	No control
Blanco et al. Cancer, [24]	Any cancer type *N* = 140 (29 vs. 111)	Candidate gene (2 SNP in 2 genes)	CHF	CBR3	rs1056892	GG/ AA	5.63 (0.80–39.57)	0.083	No control
						GA/ AA	3.66 (0.64–21.07)	0.15	
				NQO1	rs1800566	CC/ TT	1.26 (0.14–11.39)	0.84	
						CT/ TT	0.65 (0.06–6.77)	0.72	
Hildebrandt et al. Nature, [32]	Any cancer type *N* = 108 (46 vs. 62)	Candidate gene (12 SNP)	Cardiotoxicity	PLCE1	rs932764	G	0.48 (0.27–0.85)	0.012	No control
				ATP2B1	rs17249754	G	0.33 (0.12–0.92)	0.034	
				PLCE1	rs932764	G	0.36 (0.18–0.76)	0.0068	
				ATP2B1	rs17249754	G	0.26 (0.07–0.96)	0.04	
Aminkeng et al. Nat Genet., [23]	Any cancer type *N* = 376 (54 vs. 322)	GWAS	ACT	RARG	rs2229774		4.7 (2.7–8.3)	5.9 × 10^−8^	Bonferroni correction
				RARG	rs2229774	AA+AG/ GG	5.2 (3.0–9.0)	5.9 × 10^−10^	
Visscher et al. Pharmacogenomics, [22]	Any cancer type *N* = 335 (78 vs. 257)	GWAS	ACT	SLC22A17	rs4982753	A/ G	0.52 (0.31–0.85)	0.0078	Bonferroni correction
				SLC22A7	rs4149178	G/ A	0.41 (0.21–0.77)	0.0034	
Wang et al. JCO., [27]	Any cancer type *N* = 331 (112 vs. 219)	GWAS	Cardiomyopathy	CELF4	rs1786814	GA/ AA	3.18 (0.5–18.9)	0.2	Multiple testing correction
						GG/ AA	6.73 (1.1–41.1)	0.04	
						GG/AA+GA	2.26 (1.2–4.0)	0.006	
Wang et al. JCO, [26]	Any cancer type *N* = 287 (93 vs. 194)	GWAS	Cardiomyopathy	HAS3	rs2232228	GA/ GG	2.0 (0.8–4.6)	0.12	Multiple testing correction
				HAS3	rs2232228	AA/ GG	1.8 (0.7–4.7)	0.2	
Visscher et al. JCO, [20]	Any cancer type *N* = 156 (38 vs. 118)	GWAS	ACT	SLC28A3	rs7853758	A	0.31 (0.16–0.60)	1.0 × 10^−4^	simpleM correction
Chaix et al. JACC : Cardiooncology, [30]^a^	Any cancer type *N* = 289(183 vs. 106)	WES	Cardiotoxicity	CELF4			0.18 (0.06–0.56)	<0.001	Bonferroni correction
				ZNF71			0.05 (0.00–0.92)	<0.001	
				PIK3R2			0.05 (0.00–0.92)	<0.001	
Sharafeldin et al. JACC: Cardiooncology, [39]	Any cancer type *N* = 278 (129 vs. 149)	WES	Cardiomyopathy	TNIK			4.58 (2.47–8.49)	1.34 × 10^−6^	Bonferroni correction
				NOBOX			7.21 (3.23–16.1)	1.43 × 10^−6^	
				LRRK2			0.19 (0.09–0.39)	6.62 × 10^−6^	
				P2RX7			0.10 (0.04–0.27)	2.19 × 10^−6^	
				MEFV			0.08 (0.03–0.24)	4.07 × 10^−6^	
				FBN3			4.59 (2.42–8.71)	3.05 × 10^−6^	
Krajinovic et al. The Pharmacogenomics Journal, [33]	ALL *N* = 251	Candidate gene (33 common SNP in 12 genes)	LVEF	ABCC5	rs7627754	TT/ AA+AT	−12%^d^	<0.0005	FDR control
			LVFS	ABCC5	rs7627754	TT/ AA+AT	−8%^d^	0.001	
Semsei A et al. Cell Biol. Int, [34]	ALL *N* = 235	Candidate gene (9 SNP in 1 gene)	LVFS at the last follow-up time	ABCC1	rs246221	TT/ CC/ TC	38.5%/ 40.7%/ 38.4%^c^	0.027	Bonferroni correction
Lipshultz S et al. Cancer. [35]	High risk ALL *N* = 184	Candidate gene (2 SNP in 1 gene)	LVSF 4 to 7 years after diagnosis	HFE	Combined H63D/C282Y	Carriers	−0.93% (0.27)^e^	0.003	No control
Petrykey et al. Pharmacogenomics, [37]	ALL *N* = 233	WESc^b^	LVEF	TTN	rs72648998	CC/ CT+TT	4.78 (2.4–7.3)^e^	0.0003	FDR control
				TTN	rs3829747	CC/ CT+TT	3.33 (1.5–5.2)^e^	0.0003	
				TTN	rs2303838	CC/ CT+TT	2.59 (0.9–4.2)^e^	0.0005	
			LVFS	TTN	rs72648998	CC/ CT+TT	3.35 (1.6–5.1)^e^	0.0002	
			LVEF	NOS1	rs76090928			0.0013	
				ABCG2	rs199473672			0.0026	
Sapkota et al. JNCI, [36]	Any cancer type *N* = 1870 (227–1643)	WGS	CCD	KCNK17	rs2815063	A	1.57 (1.19–2.09)	0.002	FDR control
			LVEF	KCNK17	rs2815063	A	−0.016 (0.003)^e^	2.1 × 10^−8^	
					rs115537302	A	−0.019 (0.004)^e^	8.7 × 10^−8^	
					rs115057884	C	−0.019 (0.004)^e^	9.0 × 10^−8^	
					rs116181858	G	−0.019 (0.004)^e^	9.6 × 10^−8^	
					rs114566901	C	−0.019 (0.004)^e^	1.7 × 10^−7^	
Sapkota et al. AACR, [29]	Any cancer type *N* = 246 (40 vs. 206)	WGS	LVEF	1p13. 2	rs6689879	C/ T	−0.042 (0.007)^e^	2.8 × 10^−8^	Multiple testing correction
				15q25. 3	rs9788776	G/ A	−0.059 (0.01)^e^	3.5 × 10^−8^	
			Cardiomyopathy (grade 2–4)	1p13. 2	rs6689879	C/ T	3.71 (1.87–7.37)	1.8 × 10^−4^	
				15q25. 3	rs9788776	G/ A	5.24 (2.30–11.92)	7.9 × 10^−5^	

*ALL* acute lymphoblastic leukemia, *OR* odds ratio, *CI* confidence interval, *SE* standard error, *ACT* anthracycline-induced cardiotoxicity, *CHF* congestive heart failure, *CCD* cancer treatment–induced cardiac dysfunction, *LVEF* left-ventricular ejection fraction, *LVFS* left-ventricular fractional shortening.

^a^28 genes significantly associated with case-control status but we only reported the 3 genes considered as the more important by the authors.

^b^Allele frequency.

^c^With a focus on genetic variants in genes selected for doxorubicin action pathways, in the functioning of the cardiac system, and in the mitochondrial function.

^d^Effect size measured by echo-cardiac parameters.

^e^Beta (SE) or Beta (95%CI).

**Table 3:** Genes and variant effect according to cumulative anthracycline doses on cardiomyopathy.

Study	Tumor type and cohort size (case vs. controls)	Outcome	Interaction or stratification	Categories definitions	Genes /variants	Effect allele	Cat 1	Cat 2	Cat 3	Correction for multiple testing
							OR (95% CI)	OR (95% CI)	OR (95% CI)	
Blanco et al. JCO, [25]	Any cancer type 487 (170 vs. 317)	Cardiomyopathy	Interaction SNP × cumulative anthracycline doses (3 categories)	Cat 1: no exposure Cat 2: 1 to 250 mg/m2 Cat 3: > 250 mg/m²	CBR3 / rs1056892	GA+AA	Ref	1.7 (0.5–5.7)	18.9 (6.1–58.4)	Bonferonni correction
						GG	0.9 (0.2–3.4)	5.5 (1.8–16.6)	25.9 (7.7–87.6)	
					CBR1/ rs9024	GA+AA	Ref	0.7 (0.1–3.6)	24.6 (5.4–112.0)	
						GG	0.4 (0.1–1.5)	2.2 (0.6–8.2)	11.2 (2.9–12.6)	
					CBR3 / rs1056892 and CBR1/ rs9024	GA+AA and GA+AA	Ref	1.5 (0.5–4.8)	17.4 (5.9–51.2)	
						GG and GG	0.5 (0.1–2.7)	5.4 (1.8–16.2)	17.9 (5.6–57.1)	
Singh et al. Cancer, [28]	Any cancer type 167 (75 vs. 92)	Cardiomyopathy	Stratification on cumulative anthracycline doses	Cat 1: ≤ 250 mg/m² Cat 2: > 250 mg/m²	GSTM1	Null / Positive	2.3 (0.7–6.7)	2.6 (1.2–5.1)		No control
Wang et al. JCO, [27]	Any cancer type 331 (112 vs. 219)	Cardiomyopathy	Interaction SNP × cumulative anthracycline doses (2 categories)	Cat 1: ≤ 300 mg/m² Cat 2: > 300 mg/m²	CELF4/ rs1786814	GA+AA*	Ref	1.8 (0.6–5.0)		Multiple testing correction
						GG	1.0 (0.5–2.2)	10.2 (3.8–27.3)		
Wang et al. JCO, [26]	Any cancer type 287 (93 vs. 194)	Cardiomyopathy	Interaction SNP × cumulative anthracycline doses (continuos)	Cat 1 : cumulative anthracyclines dose > 450 mg/m²	HAS3/ rs2232228	AA	56.5 (NA)			Multiple testing correction
						GG	0.6 (NA)			
				Cat 1: Dose > 250 mg/m²	HAS3/ rs2232228	GG	Ref			
						GA	4.7 (1.4–16.2)			
						AA	8.9 (2.1–37.5)			
			Interaction SNP × cumulative anthracycline doses (2 categories)	Cat 1: 0 –250 mg/m^2^ Cat 2: > 250 mg/m^2^	HAS3/ rs2232228	GG	Ref	1.1 (0.3–4.8)		
						GA	0.5 (0.2–1.8)	5.2 (1.6–17.4)		
						AA	0.2 (0.1–0.8)	9.9 (2.4–40.9)		
Wang et al. JCO, [38]	Any cancer type 1866 (126–1740)	Cardiomyopathy	Interaction SNP × cumulative anthracycline doses (2 categories)	Cat 1: ≤ 250 mg/m^2^ Cat 2: > 250 mg/m^2^	ROBO2/ rs17736312	GG+AG	Ref	0.14 (1.1–2.9)		Multiple testing correction
						AA	1.8 (1.1–2.9)	2.2 (1.2–4.0)		

*ACT* anthracyclines-Induced cardiotoxicity, *LVFS* left-ventricular fractional shortening, *LVEF* left-ventricular ejection fraction, *OR* odds ratio, *CI* confidence interval.

#### Case-control studies (*n* = 15)

Among the 15 case-control studies, six used a gene candidate approach, six a GWAS, and two a WES approach.

##### Candidate gene

The number of variants tested ranges from two to 70 per study. In survivors of ALL or osteosarcoma, carrying the *ABCC2*-rs3740066-GG genotype or the *SLC22A6*-rs6591722-AA genotype increased the risk of cardiomyopathy 5–10 years after cancer diagnosis compared to other genotypes (*p* = 7.11 × 10^−4^, *p* = 1.71 × 10^−3^, respectively). A similar association was observed in patients carrying the T allele in the *NQO1*-rs1043470 variant (*p* = 5.82 × 10^−3^) [31]. The *CYP3A5*-rs4646450-TT and the *SLC28A3*- rs7853758-AA genotypes were associated respectively with a 6.6-fold (95% CI: 1.7–25.7, *p* = 7.00 × 10^−3^) and 9.8-fold (95% CI: 1.7–56.0, *p* = 0.01) increased risk of cardiomyopathy [31].

In survivors treated for heterogeneous types of cancers, the *CBR3*-rs1056892-GG variant genotype showed an increased risk of developing cardiomyopathy (OR = 1.79, 95% CI: 1.08–2.96), *p* = 0.02) [25], and also showed an interaction effect with exposition to low-to-moderate doses of anthracycline (1–250 mg/m²), increasing by 3.3-fold increased risk of cardiomyopathy compared to GA and AA genotypes [25]. For both *CBR3*-rs1056892 and *CBR1*-rs9024 variants, carrying the GG genotype along with receiving low-to-moderate doses of anthracycline increased the risk of cardiomyopathy by 3.6-fold compared to AA and GA genotypes [25]. Carrying the A allele in the *SLC28A3*- rs7853758 variant was associated with a lower risk of cardiomyopathy (OR = 0.36, 95% CI: 0.22–0.60) [21], and the variant *UGT1A6*-rs17863783 was significantly associated with a 6.2-fold (95% CI: 2.5–15.4; *p* = 1.1 × 10^−4^) increased risk of severe cardiomyopathy [21]. The *GSTM1* null variant was more prevalent among cases with cardiomyopathy than controls (60.0% vs. 38.0%, *p* = 0.005): individuals with *GSTM1* null genotype had 2.7-times greater risk of developing cardiomyopathy (*p* = 0.007) compared to those with *GSTM1* positive genotype [28]. The analysis of *NQO1*-rs1800566 (NQO1*2 polymorphism) and *CBR3*-rs1056892 (V244M polymorphism) variants revealed that they were not associated with the development of CHF [24]. The *PLCE1-*rs932764-G and *ATP2B1-*rs17249754-G variants, known for their role in hypertension, were significantly associated with a decreasing risk of cardiomyopathy (*p* = 0.006 and 0.04, respectively) [32].

##### GWAS

Patients carrying *ROBO2*-rs17736312-AA genotype and exposed to anthracyclines >250 mg/m² demonstrated a 2.2-fold (95% CI: 1.2–4.0, *p* = 0.009) higher risk of CHF compared to patients carrying at least one G allele and exposed to less than 250 mg/m² of anthracyclines [38]. However, in the same study, no variant reached the threshold for genome-wide significance in their association with cardiomyopathy [38]. In the *RARG*-rs2229774 variant, carrying at least one A allele was associated with an increased risk of cardiomyopathy following anthracycline exposition compared to the GG genotype (*p* = 5.9 × 10^−10^) [23]. Variants *SLC22A17*-rs4982753 and *SLC22A7-*rs4149178 were associated with decreasing risk of cardiomyopathy in Canadian CCS (*p* = 0.0078 and 0.0034, respectively) and were validated in a replication cohort (*p* = 0.0071 and 0.047, respectively). Additionally, the variant *SULT2B1*-rs10426628 was associated with a 1.9-fold increased risk of cardiomyopathy in the combined cohort (95% CI: 1.2–2.5, *p* = 3.2 × 10^−4^) [22]. The *CELF4*-rs1786814-GG genotype was associated with an increased risk of cardiomyopathy compared to GA and AA genotypes (*p* = 0.006), and when exposed to doses lower or equal to 300 mg/m², patients carrying GG genotype were at 5.8-fold higher risk of developing cardiomyopathy compared to GA and AA genotypes [27]. In addition, exposition to anthracycline doses >250 mg/m² and carrying at least one A allele in the HAS3-rs2232228 variant were at increased risk of cardiomyopathy compared to the GG genotype (AA: *p* = 0.003 and AG: *p* = 0.02) [26]. Moreover, carrying the A allele in the *SLC28A3*-rs7853758 variant was associated with a decreased risk effect on cardiomyopathy (OR = 0.31, 95% CI: 0.16–0.60) [20].

##### WES

Twenty-eight genes carrying at least one rare to low-frequency variant were identified as associated with protective effects against cardiomyopathy. Among the controls, 89.6% harbored rare-to-low-frequency variants, while only 42.6% of patients with cardiomyopathy were carriers. The *ZNF827* and the *PI3KR2* genes showed a more significant variant burden in controls compared to patients experiencing cardiomyopathy [30]. Six genes carrying various SNPs were significantly associated with cardiomyopathy. The association between these SNP individually and the cardiomyopathy was analyzed but not presented in the study because considered to be “of minimal clinical and public health importance”. Among these six genes, the P2RX7 gene showed two complementary variants (rs208294 and rs3751143) whose union, having one or no copies of variant yielded a reduced risk of cardiomyopathy in CCS (OR = 0.10; 95% CI: 0.04–0.27; *p* = 2.19 × 10^−6^). These findings were replicated the in an independent cancer survivor population [39].

#### Cohort studies (n = 5)

Among the five cohort studies, three used a gene candidate approach, two a WGS, and one a WES. The outcomes for these five studies were LVEF or LVFS; four studies investigated cohorts of ALL survivors, and only one worked on a cohort of CCS treated for heterogeneous first malignancies.

##### Candidate gene

In ALL survivors, the *ABCC5*-rs7627754-TT genotype was associated with a 12% reduction LVEF and 8% reduction of LVFS. However, prior to cancer treatment exposure, carriers of the TT genotype already had lower LVEF and LVFS values compared to other genotypes [33]. A protective effect of the *NOS3*-rs1799983 variant was observed in patients carrying the TT genotype, who presented a higher LVEF than individuals with at least one G allele (61.4% vs 59%, *p* = 0.02). This protective effect was more significant in high-risk patients who did not receive dexrazoxane before doxorubicin (64.1% vs. 56.8%, *p* = 0.002) but was not observed in patients who received that treatment [33]. Carrying TT genotypes for the *ABCC1*-rs3743527 variant was associated with a reduced LVFS at the end of treatment (34.0%), compared to CC (39.5%) or CT (39.3%) genotypes (*p* = 0.001). In addition, patients with at least one T allele in the *ABCC1*-rs246221 variant showed a lower LVFS than patients with homozygous C alleles (Allele frequency: TC: 38.4%, TT: 38.5%, CC: 40.7%, *p* = 0.027) [34]. After anthracycline administration, the mean LVFS was significantly lower in patients with a combination of *ABCC1*-rs3743527-TT and *ABCC1*-rs246221-TC or TT genotypes compared to patients carrying other genotypes (*p* = 0.001) [34]. Compared to a population of healthy children, high-risk ALL patients with the C282Y and/or H63D allelic variants in the *HFE* gene had significantly lower LV function, mass, and wall thickness two years after their diagnosis [35].

##### WES

Common variants analysis revealed that the genotypes *TTN*-rs72648998-CC and *TTN*-rs3829747-CC were associated with an increased LVFS (*p* = 0.0002 and 0.0004, respectively) and LVEF (*p* = 0.0003 and 0.0003, respectively), and that *TTN*-rs2303838-CC genotype was associated with an increased LVEF (*p* = 0.0005) [37]. Rare variants *NOS1*-rs76090928, *ABCG2*-rs199473672, *ABCG2-*rs45605536, *AKR1C3*-rs200981816, and *AKR1C3*-rs34186955 were associated with a protective effect on LVEF, and a threatening effect was observed for the *CBR1*-rs2835266 variant [37]. In the high-ALL risk group, the ABCC5 gene had a protective effect on LVEF and LVFS (*p* = 0.0008 and *p* = 0.0014, respectively). In addition, in survivors treated according to the high-ALL risk protocols, the rare variant enrichment in the ABCC5 gene was associated with a protective effect on LVEF and LVFS [37]. An association between LVFS and rare variants enrichment in the *NOD2* and *ZNF267* genes (*p* = 1.39 × 10^−6^ and 3.51 × 10^−6^, respectively) was detected. An association was also observed between rare variant enrichment in *NOD2* and LVEF (*p* = 2 × 10^−6^) [37].

##### WGS

The *KCNK17*-rs2815063-A allele was associated with a reduced LVEF in patients diagnosed with a cardiomyopathy grade ≥3 after adjusting for cardiac medication (*p* = 7.5 × 10^−6^). After classification into therapy-based risk groups, the association between *KCNK17*-rs2815063-A and LVEF reduction was stronger in the high and moderate cardiomyopathy risk groups (*p* = 9.6 × 10^−4^ and *p* = 1.4 × 10^−4^, respectively), compared to the low-risk group (*p* = 0.002). This association was also replicated in individuals with African ancestry (*p* = 0.004) [36]. In a secondary case-control analysis, the same author observed a 1.4-fold increased risk of CHF (95% CI: 1.02–1.92, *p* = 0.004) in individuals carrying the *KCNK17*-rs2815063 variant with the A allele. This polymorphism also revealed an elevated risk of cardiac events with OR = 1.6 (95% CI: 1.19–2.09) in grade ≥2 cardiomyopathy and OR = 1.8 (95% CI: 1.17–2.77) in grade ≥3 cardiomyopathy [36].

In CCS with African ancestry, 31 sets of rare/ low-frequency variants and two loci (1p13.2 and 15q25.3) in the common variant analysis (rs6689879 and rs9788776, respectively) were significantly associated with LVEF [29]. The rs6689879-C and rs9788776-G alleles were significantly associated with a reduced LVEF (*p* = 2.8 × 10^−8^ and *p* = 3.5 × 10^−8^, respectively), and also showed a reduction effect in patients exposed to anthracyclines only. However, in patients exposed to RT only, the rs9788776-G allele showed a reduction of 8.1% of the LVEF (*p* = 5.4 × 10^−4^) [29]. These two variants were not associated with LVEF in patients not exposed to cardiotoxic treatments (anthracyclines and chest-RT).

## Discussion

This systematic review identified 20 studies investigating the associations between genetics and cardiomyopathy in CCS, 15 of which (75%) conducted case-controls, while five (25%) conducted cohort studies. Several genetic variants in 69 genes were identified as significantly associated with cardiomyopathy, using diverse sequencing methods.

Among these variants, *SLC28A3*-rs7853758, *RARG*-rs2229774, and the union of *P2RX7*-rs208294 and *P2RX7*-rs3751143 variants represent the most consistent findings, showing strong and significant associations with cardiomyopathy following childhood cancer treatment, including anthracycline; these associations have been successfully replicated in patients from external cohorts [20, 21, 23, 39]. The rs7853758 variant in the *SLC28A3* gene has been identified as protective against cardiomyopathy. This gene is known to contribute to the transport of anthracyclines across cell membranes and their elimination [40, 41]. This suggests that the elimination of anthracyclines from cardiac may be more efficient in CCS who carry of the *SLC28A3*- rs7853758 variant. A candidate gene study on early breast cancer patients that focused on the *SLC28A3* gene found no cardiomyopathy association after anthracycline treatment [42], suggesting a potential specificity of this gene to CCS. Patients carrying the *RARG*-rs2229774 variant have an increased risk of cardiomyopathy ; the *RARG* gene is involved in the transcriptional regulation of an enzyme, Topoisomerase-2β, which is involved in DNA replication and repair, and that interacts with doxorubicin in cardiac tissues, leading to cardiomyocyte apoptosis and heart failure in mice [14]. This variant may inhibit the transcription of Topoisomerase-2β, potentially contributing to the development of cardiac dysfunction. Carrying one or no copy of the *P2RX7-*rs208294 and *P2RX7*-rs3751143 variants, is associated with a reduced risk of cardiomyopathy. The *P2RX7* gene is involved in cell apoptosis mechanism, and is activated by high levels of extracellular ATP which opens cationic channels facilitating the flow of small cations, such as Ca^2+^ [43], and calcium overload leads to sarcomere damages, followed by cardiomyocyte death [44]. The underlying hypothesis is that the simultaneous presence of these two variants would lead to calcium overload and cardiomyocyte death, resulting in long-term cardiac dysfunction.

In addition to these main variants, our review also identified numerous other genes and variants that require further validation, and highlighted interactions between genes/variants and cumulative doses of anthracyclines, as well as ethnic and gender-specific genes/variants. However, too few studies have investigated the excess risk of RT doses to the heart on the development of CD in CCS, and furthermore, no study has investigated the existence of interactions between identified genes/variants and RT. These other genes/variants were implicated in various biological mechanisms, first among them, the genes involved in drug transport, accumulation, and/or elimination. Different variants in the Solute carrier (SLC) transporters: *SLC28A3*, *SLC22A17*, *SLC22A7*, and *SLC22A6* were identified as associated with cardiomyopathy in CCS. An association with an increased risk of cardiomyopathy has also been observed for the *SULT2B1* gene, which contributes to the elimination of drugs such as anthracyclines and their toxic metabolites [45]. The rs17863783 variant in the *UGT1A6* gene was also reported to be associated with a greater risk of cardiomyopathy. This variant encodes for an enzyme that eliminates various drugs, and is known to impair the ability of this enzyme to effectively eliminate anthracyclines [14]. Also a cardiotoxicity effect was observed for genes from the ABC transporters group of genes, specifically subfamilies B and C, which transport xenobiotics like anthracyclines, and whose inhibition disrupts the distribution and metabolism of antineoplastic agents, leading to increased plasma and tissue concentrations of anthracyclines [46].

An important biological mechanism reported in the included studies is oxidative stress induced by anthracyclines, resulting in an increased formation of reactive oxygen species (ROS), leading to a reduction of cardiac contractility, tumor cell apoptosis, and cardiomyocyte damage [47–50]. Different genes involved in this mechanism were reported, such as the *PLCE1* gene, which encodes for a cardioprotective protein known for its effect against oxidative stress; *NQO1*, which participates in the anthracyclines reduction and prevents the formation of ROS [48]; *NOS1* and *NOS3*, which catalyzes the nitric oxide production that contributes to anthracycline cardiac damage and the production of ROS [14, 48]; or *HAS3*, which encodes for the synthesis of an extracellular matrix known to capture, and thus reduce, ROS [51]. Also, the ROS generation induces the expression of *GSTM1* to cancel cellular oxidative stress caused by anthracycline exposure [48, 50], which explains how the complete deletion of the *GSTM1* gene (*GSTM1* null variant) is associated with cardiomyopathy after anthracycline treatment. Other genes, not related to any of the biological mechanisms mentioned above, were also identified in this review, like the *HFE* gene involved in iron metabolism [14] and the *TTN* gene, whose protective effect contradicts a priori knowledge on its role in heart disease [52].

Protein and enzyme regulation is also an important mechanism reported in this review, including the *CELF4* gene, which the mRNA splicing of a protein (Troponin 2) that enables cardiac muscle contraction [14]. In addition, two ethnically specific variants were also identified in individuals with African ancestry - rs9788776 and rs6689879 - with findings not replicated in the cohort of patients with European ancestry because of the monomorphic nature of rs9788776 in this population and of the insignificant association of rs6689879 in these patients [29].

One previous review [17] investigated genetic predispositions to anthracycline-induced cardiotoxicity, but was not specific to pediatric cancers which are histopathologically and molecularly different from adult cancers. It is, therefore, imperative to focus on CCS in order to build a complete list of genes involved in pediatric malignancies and cancer-treatment late effects, and to develop more effective and less toxic treatments [53, 54]. In this sense, Aminkeng, et al. [55] proposed recommendations for using pharmacogenomic testing to reduce cardiomyopathy incidence in patients treated for cancer, but did not specifically address CCS. Indeed, it would be interesting to add a genetic screening to the clinical guidelines for patients diagnosed with childhood cancer targeting the *RARG*- rs2229774 risk variant, the *SLC28A3*-rs7853758 protective variant or the harmful union of the *P2RX7*-rs208294 and *P2RX7*-rs3751143 variants. Screening upstream of cancer treatment could improve the identification of patients at risk of developing CD. In the first stage, we could try to tailor the treatment of high-risk patients by administering cardioprotective treatments during cancer treatment. In the second stage, after cancer treatment, to tailor the long-term follow-up protocol for these patients who are at increased risk of developing cardiac pathology, by proposing rigorous follow-up and more frequent echocardiography than low-risk patients.

The review of Berkman et al. [18] was focused on anthracycline-induced cardiotoxicity, and did not address the effects of RT involving the heart. Although our review aimed introduce the impact of RT in CD development, only 15 studies (75%) reported treatment with RT involving the heart, and none investigated interactions between the identified genes and this cancer treatment even though the risks of CD associated with RT involving the heart are now well known. This limitation can be explained by the fact that CD associated with RT involving the heart take several decades to manifest. Indeed, in the absence of anthracycline exposure, around 25% of patients exposed to average radiotherapy doses to the heart >15 Gy will develop cardiac pathology by age 40 [4]. In this systematic review, cardiomyopathy was the only outcome identified, and is more often associated to anthracyclines exposure than RT. Moreover, information on the radiation dose received to the heart may be difficult to obtain when the irradiated organ is distant from the heart. Nevertheless, tools that estimate the dose-volume distribution of radiation in the whole heart and the substructures of the heart are currently available [4, 9, 56], and could enhance the collection of dosimetry information involving the heart. Additionally, CD associated to RT, such as coronary artery diseases, myocardial and pericardial fibrosis, or valvular diseases, are physiologically different from those associated to anthracyclines [57] and are less common than cardiomyopathy, establishing international collaborations would be relevant in order to have sufficient number of events to conduct robust analyses. Future directions must focus on interactions between treatment-related risk factors, radiation doses and volume metrics - considering the different cardiac substructure - anthracycline and other chemotherapy doses with genetic predisposition to CD.

As described in the current review, the high number of identified variants and genes makes it difficult to draw firm conclusions. Despite effort to reduce heterogeneity between the studies included in the systematic review, it would be interesting to access and analyse individual patient data in meta-analyses. This would make it possible to assess heterogeneity between studies and perform subgroup and sensitivity analyses. Additionally, focusing on rare or ultra-rare genetic variants might reveal a more important effect than common variants in CCS. Also, opening up new fields of research, such as genes expression, could be an interesting: the dynamic nature of the transcriptome, responsive to xenobiotics, allows the identification of biomarkers that can directly be used for detection of pathologies resulting from exposure to treatments [58]. It is also important not to focus solely on cardiomyopathies, but to study all cardiac diseases, which may have different pathophysiological mechanisms involving different genes. Studies in these different fields require the inclusion of thousands of CCS and can, therefore, only be carried out through international collaboration. This would produce the robust results that could be considered in the international guidelines of patients treated for childhood cancer. Indeed, current CCS follow-up guidelines do not include genetic screening [59–61], even though a number of studies have shown the benefits of including genetic variables in statistical models comprising clinical variables by improving the discriminative accuracy of the models, and allowing a better distinction between patients who developed cardiomyopathy and those who did not [20–22].

In conclusion, this systematic review presents an overview of the genetic variants potentially predisposing to cardiomyopathy, and provides further support for the important role of the four main variants in risk stratification of CD in CCS. Indeed, it is crucial to prevent late effects, such as cardiac disease, and to improve CCS management. Despite therapeutic advances, CCS are still at increased risk of developing late health concerns. It would be extremely useful to establish a set of gene variants and biomarkers, in order to identify patients most at risk of developing CD, and to adapt as much as possible their exposition to cardiotoxic therapies, as well as to implement monitoring and prevention.

## Supplementary information

Supplementary Table 1

Supplementary Table 2

Supplementary Table 3

Supplementary Table 4

Appendix

## Data Availability

The data underlying this article will be shared on request to the corresponding author.
