# Supplementary material for: Influence of genetic biomarkers on cardiac diseases in childhood cancer survivors: a systematic review
Source: Pharmacogenomics J. 2025 May 24;25(3):15. doi: 10.1038/s41397-025-00369-y (PMC12103300; doi:10.1038/s41397-025-00369-y)
Supplement: Supplementary file 1 — Supplementary Table 1 [file 41397_2025_369_MOESM1_ESM.docx]

**Supplementary Table 1:** Details of treatments of the 20 included studies

| Study | Anthracycline | Radiotherapy | Other treatments |
| --- | --- | --- | --- |
| Sági et al., BMC Cancer, 2018 | All patients were treated with anthracyclines, Cumulative anthracycline exposure calculated using doxorubicin equivalent.  ALL low-risk and medium-risk groups of doses between 180 and 240 mg/m².  ALL high-risk group and relapsed patients: doses between 240 and 380 mg/m².  Osteosarcoma standard-risk group: 360 mg/m²  high-risk group: 180 mg/m². | NA | Treatment protocols:  ALL BFM (Berlin–Frankfurt–Münster) 88, ALL BFM 90, ALL BFM 95, ALL IC-BFM (ALL Intercontinental) 2002 or ALL IC-BFM 2009; Interfant 98 or Interfant 2006.  COSS (German-Austrian-Swiss osteosarcoma study group) -86 and COSS-96 protocols.  29% of the patients received 12 Gy cranial radiotherapy. |
| Blanco et al., JCO, 2012 | Cumulative anthracycline exposure calculated using a factor that reflects the drug’s cardiotoxic potential (mg/m²).  Cumulative anthracycline exposure was treated as a categorical variable (0, 1- 100, 101 - 150, 151 - 200, 201- 250, 251 - 300, > 300). | Radiation therapy to the chest including the heart: (Yes/No) variable. | NA |
| Visscher et al., Pediatr Blood Cancer, 2013 | All Canadian patients received anthracyclines.  Cumulative anthracycline doses were calculated using doxorubicin equivalents.  Cumulative anthracycline exposure was treated as a continuous variable. | Radiotherapy to the heart: (Yes/No) variable. | NA |
| Singh et al., Cancer, 2020 | Cumulative anthracycline exposure calculated using a factor that reflects the drug’s cardiotoxic potential (mg/m²).  Cumulative anthracycline exposure was treated as a continuous variable. | Radiation therapy to the chest including the heart: (Yes/No) variable. | NA |
| Blanco et al., Cancer. 2008 | Cumulative doses of anthracyclines. | Radiation to the heart: total cumulative dose in cGy to the heart. | NA |
| Hildebrandt et al., Nature, 2017 | Cumulative anthracyclines dose was standardized to doxorubicin equivalents. | NA | All patients who received anthracycline-based chemotherapy were also treated with other agents as indicated by cancer diagnosis.  Some patients were treated with cardiac medication. |
| Aminkeng et al., Nat Genet. 2015 | All patients received anthracyclines.  Cumulative anthracycline doses were calculated using doxorubicin equivalents.  Cumulative anthracycline exposure was treated as a continuous variable. | Radiation therapy included significant radiation exposure to the heart or surrounding tissue: mantle and mediastinal radiation, whole-lung radiation, whole-abdomen or upper abdominal radiation, left-side flank radiation, and total-body irradiation: (Yes/No) variable. | NA |
| Visscher et al., Pharmacogenomics, 2015 | All Canadian patients received anthracyclines.  Cumulative anthracycline doses were calculated using doxorubicin equivalents.  Cumulative anthracycline exposure was treated as a continuous variable. | Radiotherapy to the heart: (Yes/No) variable. | NA |
| Wang et al., JCO, 2016 | Cumulative anthracycline exposure calculated using a factor that reflects the drug’s cardiotoxic potential (mg/m²)  Cumulative anthracycline exposure was treated as a continuous variable. | Radiation therapy to the chest including the heart: (Yes/No) variable. | NA |
| Wang et al., JCO, 2014 | Cumulative anthracycline exposure calculated using a factor that reflects the drug’s cardiotoxic potential (mg/m²)  Cumulative anthracycline exposure was treated as a continuous variable. | Total radiation therapy to the chest including the heart: continuous variable. | NA |
| Wang et al., JCO, 2022 | Anthracycline exposure included exposure to doxorubicin, daunorubicin, idarubicin, epirubicin, or mitoxantrone.  Cumulative anthracycline exposure calculated using a factor that reflects the drug’s cardiotoxic potential (mg/m²). | Radiation to the chest with heart in the field was captured as a yes/no variable. | NA |
| Visscher et al., JCO, 2012 | All Canadian patients received doxorubicin or daunorubicin.  23/96 patients in the Dutch-EKZ cohort received other anthracyclines instead.  Cumulative anthracycline doses were calculated using doxorubicin equivalents.  Cumulative anthracycline exposure was treated as a continuous variable. | Radiotherapy to the heart: (Yes/No) variable. | NA |
| Chaix et al., JACC : Cardiooncology, 2020. | The anthracycline cumulative dose was measured in doxorubicin equivalents (mg/m²).  Cases received low cumulative anthracycline dose (≤250 mg/m^2^)  Controls received high dose anthracycline (>250 mg/m^2^). | Radiation therapy to the chest including the heart: (Yes/No) variable. | Dexrazoxane |
| Sharafeldin et al., JACC: Cardiooncology, 2023 | Anthracycline exposure was calculated by multiplying the cumulative dose (mg/m²) of individual anthracyclines (doxorubicin, daunomycin, epirubicin, or idarubicin) by a drug cardiotoxicity factor and summing the results. | Radiation therapy to the chest when the heart was in the radiation field: (Yes/No) variable. | NA |
| Krajinovic et al., The Pharmacogenomics Journal, 2016 | All patients received at least two 30 mg/m² doses of doxorubicin.  High-risk patients continued to receive one weekly doxorubicin dose during the consolidation phase, for a total cumulative dose of 300 mg/m² for the 95-01 and 00-01 protocols, or 360 mg/m ² for the 87-01 and 91-01 protocols. | NA | DFCI consortium 87-01, 91-01, 95-01, or 00-01.  For 95-01 protocol, high-risk patients were randomized to receive (or not) a 300 mg/m² dose of Dexrazoxane during induction and consolidation therapy.  For 00-01 protocol, all high-risk patients received dexrazoxane before doxorubicin administration. |
| Semsei A., Cell Biol. Int, 2012 | Cumulative anthracyclines doses in mg/m² | NA | Chemotherapy protocol: ALL BFM 90 or ALL BFM 95.  The chemotherapy regimen included repeated doses of intravenous vincristine, L-asparaginase, daunorubicin, doxorubicin, methotrexate, cyclophosphamide, cytosine arabinoside, oral prednisone, dexamethasone, mercaptopurine, thioguanine, intrathecal methotrexate, intrathecal prednisone and ifosfamide (the latter only in patients with high-risk leukaemia). The protocol in the low-risk and medium-risk arms differed only in the number of anthracycline doses, the cumulative anthracycline doses being between 180 and 300 mg/m2.  The high-risk arm differed considerably from the low-risk and medium-risk arms in terms of the applied therapy dosage.  Administration of dexrazoxane (Yes / No). |
| Lipshultz S., Cancer, 2013 | Total planned cumulative doxorubicin dosage: 300–360 mg/m².  Cumulative DOX dose in mg/m². | NA | ALL Consortium Protocol (1991).  Administration of dexrazoxane (Yes / No). |
| Petrykey et al., Pharmacogenomics, 2021 | All patients were given two 30 mg/m2 doses of doxorubicin.  HR patients continued to receive one weekly doxorubicin dose during the consolidation phase, for a total cumulative dose of 294.52 mg/m². | NA | HR patients treated with 95-01 protocol were randomized to receive (or not) dexrazoxane and all HR patients treated with 00-01 and 00-05 protocols, received dexrazoxane before doxorubicin administration. |
| Sapkota et al., JNCI J Natl Cancer Inst, 2022 | Cumulative anthracycline exposure calculated using doxorubicin equivalent (mg/m²). | Mean heart radiation dose in cGy. | NA |
| Sapkota et al., AACR, 2021 | Cumulative anthracycline exposure calculated using doxorubicin equivalent (mg/m²). | Average radiation dose in Gy. | NA |

Abbreviations: BFM: Berlin-Frankfurt-Münster; DFCI: Dana-Farber Cancer Institute; Gy, Gray; ALL, Acute lymphocytic leukemia, NA, Not Available.
