# Supplementary material for: Influence of genetic biomarkers on cardiac diseases in childhood cancer survivors: a systematic review
Source: Pharmacogenomics J. 2025 May 24;25(3):15. doi: 10.1038/s41397-025-00369-y (PMC12103300; doi:10.1038/s41397-025-00369-y)
Supplement: Supplementary file 2 — Supplementary Table 2 [file 41397_2025_369_MOESM2_ESM.docx]

**Supplementary Table 2:** Sequencing techniques of the 20 included studies

| Study | biological specimens | Technique |
| --- | --- | --- |
| Sági et al., BMC Cancer, 2018 | Blood | Based on the scientific literature, 70 single nucleotide polymorphisms (SNPs) in 26 genes were selected and genotyped.  Genotyping 63 of the SNPs was conducted using TaqMan® OpenArray™ Genotyping following the manufacturer’s instructions at the Department of Medical Chemistry, Molecular Biology and Patho-biochemistry, Semmelweis University. Other 7 SNPs were genotyped using KBioscience Competitive Allele-Specific Polymerase chain reaction-on-Demand pre-validated on 7900HT Fast Real-Time PCR System. The genotyping was unsuccessful in the case of three SNPs. |
| Blanco et al., JCO, 2012 | Blood, saliva or buccal swabs | The CBR3 V244M and CBR1 1096GA polymorphisms (rs1056892, rs9024) were analyzed using validated assays for allelic discrimination with specific fluorescent probes. Laboratory personnel were blinded to case-control status. |
| Visscher et al., Pediatr Blood Cancer, 2013 | blood, saliva, or buccal swabs | Twenty-three SNPs were selected that previously showed evidence of association (p< 0.01) with ACT.  DNA samples were genotyped using a custom 96-plex Illumina Veracode GoldenGate SNP genotyping assay. This assay included an additional 63 non-study SNPs used for quality control purposes only. All SNP genotypes were manually clustered using Illumina GenomeStudio software. One SNP in FMO2 (rs2020870) could not be reliably clustered and was therefore removed from further analyses. |
| Singh et al., Cancer, 2020 | Blood | DNA and RNA were isolated using the Gentra Puregene Blood Kit and the PAXgene Blood RNA kit (both from Qiagen Inc), respectively. The concentration was measured on Nanodrop ND-1000 Spectrophotometers (Thermo Fisher Scientific). DNA integrity was analyzed by using the Quant-iT PicoGreen dsDNA Assay Kit (Thermo Fisher Scientific). RNA quality was checked using a Bioanalyzer Nanochip (Agilent Technologies), and samples with RNA integrity numbers >7 were used for microarray analysis.  GSTM1 deletion was examined by multiplex PCR using the B2M gene as control.  Illumina HumanHT-12 v4.0 Expression BeadChips (Illumina, Inc) were used for mRNA transcription.  Expression of the GSTM1 gene was measured using qrt-PCR.  For RNA sequencing of blood, libraries were prepared using the TruSeq RNA Sample Preparation Kit, according to the protocols recommended by the manufacturer. Each library was paired-end sequenced (2 × 50 bp) by using the TruSeq SBS Kit v4-HS, on a HiSeq2000 platform.  For RNA sequencing of cardiomyocytes, gene expression levels and exon use were estimated using the featureCounts function in the Subread software. |
| Blanco et al., Cancer. 2008 | Buccal cell | The NQO1*2 polymorphism [rs1800566] was examined with a validated PCR-restriction fragment length polymorphism technique. The CBR3 V244M polymorphism (rs1056892) was analyzed with a validated assay for allelic discrimination with specific fluorescent probes. |
| Hildebrandt et al., Nature, 2017 | Blood | Twelve index genetic variants were selected for genotyping based on previous evidence of significant association with hypertension from a GWAS involving >200 000 individuals in the general population. TaqMan Genotyping Assays were available for 11 of the 12 loci. Genotyping was performed according to standard protocols on the ABI 7900HT platform. All assays were performed blinded to the cardiotoxicity status of the individuals.  RNA sequencing of cardiomyocytes: RNAseq was performed in MD Anderson’s Sequencing and Microarray Facility using the Illumina TruSeq Stranded Total RNA Library Prep Kit with Ribo-Zero Gold. The 13 strand-specific libraries were pooled and divided across two lanes for sequencing with the Illumina HiSeq. 3000. Demultiplexed reads of ~100 bp length were generated for each sample. |
| Aminkeng et al., Nat Genet. 2015 | Not Specified | Discovery cohort and genotyped on the Illumina Infinium HumanOmniExpress panel (~740,000 markers), genotypes were called with the Illumina GenomeStudio software package, and SNPs were clustered using the Illumina 740K cluster file. |
| Visscher et al., Pharmacogenomics, 2015 | blood, saliva or buccal swabs | DNA samples were genotyped for 4536 SNPs using a customized Illumina GoldenGate SNP genotyping assay, designed to capture the genetic variation of over 300 key drug biotransformation genes.  In addition, tagSNPs were included that were identified using the ldSelect algorithm to select a maximally informative set of tagSNPs to assay the candidate genes.  In addition, the main SNP panel was supplemented with a custom 96-SNP Illumina Veracode GoldenGate genotyping assay to include both functional and tagSNPs in genes involved in the metabolism of anthracyclines into alcohol metabolites, as well as other SNPs possibly related to ACT that were not included in the main panel.  All SNP genotypes were manually clustered using Illumina GenomeStudio software. |
| Wang et al., JCO, 2016 | Blood, saliva or buccal swabs | Genotyping was performed on the Illumina HumanOmniExpress-12 v1.0 DNA analysis bead-chip. The final data set included 583 748 autosomal SNPs.  Significant SNP(s) identified in discovery were genotyped in the replication set by using Sequenom iPLEX SNP chemistry on a MassArray system.  Presence of SNP(s) of interest in cardiac DNA was investigated with TaqMan genotyping assays per the manufacturer’s guidelines. Splicing isoforms were amplified by nested PCR. |
| Wang et al., JCO, 2014 | Blood, saliva or buccal swabs | Genotyping was performed on the Illumina IBC cardiovascular SNP array. The final data set retained 34 912 autosomal SNPs. Significant SNP(s) identified in the discovery stage were genotyped by using Sequenom iPLEX SNP chemistry on a MassARRAY system.  Significant SNPs validated in the replication stage were genotyped with TaqMan genotyping assays. Total RNA from heart tissue samples was reverse transcribed and amplified by using one-step QuantiTect SYBR Green rt-PCR kits. Relative hyaluronan synthase 3 (HAS3) mRNA levels were obtained after normalization to reference gene (ACTB). |
| Wang et al., JCO, 2022 | Blood or saliva in the replication set | Germline DNA from 5,739 CCSS participants was genotyped on HumanOmni5Exome arrays. Imputation on the basis of the 1000 Genomes Project release v.3 reference haplotypes using IMPUTE v.2.3.0 yielded 26 135 904 high-quality SNPs and small insertions or deletions.  In the replication cohort, genotyping was performed on the Juno system according to manufacturer’s instructions. End point fluorescence values were measured on the BioMark HD system, and the Fluidigm SNP Genotyping Analysis software program was used to generate genotyping calls for each sample.  RNA concentration was measured using Nanodrop ND-1000. RNA quality was checked on Bioanalyzer Nanochip and samples with RNA integrity number.>7 were submitted to the Genomic Services Laboratory at HudsonAlpha Institute for Biotechnology, Huntsville, AL. Poly-adenylated RNAs were isolated using NEBNext Magnetic Oligo d(T)25 beads. Libraries were prepared using the TruSeq RNA Sample Preparation Kit (Illumina Inc). |
| Visscher et al., Journal of Clinical Oncology, 2012 | Not Specified | DNA samples from the two Canadian cohorts were genotyped for 2 977 SNPs using a customized Illumina GoldenGate SNP genotyping assay, designed to capture the genetic variation of 220 key drug biotransformation genes.  After quality control, 1 931 SNPs remained for analysis. DNA samples from the Dutch-EKZ replication cohort were genotyped for rs7853758 by TaqMan SNP genotyping. |
| Chaix et al., JACC : Cardiooncology, 2020. | Blood | WES was performed (average: 100 x depth) by using the Illumina HiSeq X platform. High quality paired end reads (2 x 150bp) were mapped to the human genome reference sequence by using the bwa mem aligner, version 0.7.8, and variants were called using the Genome Analysis Toolkit, version 3.8.0. |
| Sharafeldin et al., JACC: Cardiooncology, 2023 | Blood or saliva | WES with 100X coverage was performed at the HudsonAlpha Institute for Biotechnology Genomic Services Discovery Laboratory (Huntsville, AL, USA) using Illumina NovaSeq (100 base-paired-end sequencing).  For the COG and BMTSS replication sets, SNP assays were designed and ordered using Fluidigm®D3™ assay design. Genotyping was performed on the Juno™ system (Fluidigm, San Francisco, CA, USA) and 96.96 Genotyping IFCs. For the CCSS replication set, germline DNA from 5,739 participants was genotyped on Illumina (San Diego, CA) HumanOmni5Exome array |
| Krajinovic et al., The Pharmacogenomics Journal, 2016 | Not Specified | Thirty-three common polymorphisms (with minor allele frequency, MAF, higher than 5%) in 12 genes (ABCC1, ABCC2, ABCC5, ABCB1 ABCG2, NFKB1, NQO1, NOS3, MLH1, MLH2, GSTM1 and GSTT1) involved in DOX metabolic and functional pathways7 were genotyped.  Genotyping of ABCC5 and NOS3 gene polymorphisms was not decribed in the text. |
| Semsei A., Cell Biol. Int, 2012 | Blood in patients in remission; bone marrow smears, neonatal Guthrie spots, stored buffy coats in patients who were deceased before sample collection | SNPs (single nucleotide polymorphisms) were selected prioritized on the basis of their estimated functionality in this order: non-synonymous SNPs, SNPs in promoter and 39-UTR (39untranslated region) region, synonymous SNPs and intronic SNPs. The goal was to cover every haplotype block in the gene defined by the Haploview 4.1 software with 1 or 2 SNPs.  The ABCC1 rs45511401 genotypes were determined by multiplex single base extension using a SNaPshot Multiplex Kit followed by mini sequencing on an ABI 310 genetic analys. All other ABCC1 SNPs were genotyped using the GenomeLab SNPstream genotyping platform according to the manufacturer’s instructions. |
| Lipshultz S., Cancer, 2013 | Blood | Isolated DNA was prepared using NucleoSpin DNA isolation kits (BD Biosciences Clontech) in all samples at the time of mononuclear cell fraction isolation.  The two most common HFE alleles associated with hemochromatosis, were detected by directly sequencing genomic DNA with a commercial, clinically validated kit (Biotage Inc.; PyroMark HFE Cat #40-0053) run on a Pyrosequencer instrument (PSQ HS 96, Biotage Inc.) or by Sequenome and Taqman genotyping assays performed at the Harvard Partners Center for Genetics and Genomics. |
| Petrykey et al., Pharmacogenomics, 2021 | Blood | Whole exomes were captured in solution with Agilent’s SureSelect Human All Exon 50 Mb kits and sequenced on either Life Technologies SOLiD System 4.0 (mean coverage = 40×) or Illumina HiSeq 2500 platform (mean coverage = 113.1 x) at SJUHC integrated clinical genomic center in pediatrics. Reads were aligned to the hg19 reference genome using SOLiD LifeScope software for the SOLiD samples and BWA-MEM for the samples sequenced on the Illumina system. PICARD was used to mark PCR duplicates and collect sequencing quality control metrics.  Variant calling was performed using the Haplotype Caller and quality score recalibration was performed using Variant Recalibrator, both implemented in the Genome Analysis Tool Kit (GATK). Variants were selected based on the variant quality score (VQSR = PASS) and the minimum depth of coverage (DP ≥10). The final germline variants were annotated by ANNOVAR. Only missense, nonsense and splicing common and rare variants with predicted functional impact (Sift [<0.1] and PolyPhen2 [≥0.85]) were considered. Variants were defined as rare (minor allele frequency <5%) and common (minor allele frequency ≥5%) according to the reported frequency for European populations in the 1000 Genomes and ESP6500 datasets. |
| Sapkota et al., JNCI J Natl Cancer Inst, 2022 | Blood | DNA concentration was fluorometrically measured using the Quant-it dsDNA Assay Kit and DNA integrity verified visually by agarose gel electrophoresis. Unique samples were barcoded and WGS was performed at the HudsonAlpha Institute for Biotechnology Genomic Services Laboratory using the Illumina HiSeq X10 sequencers to generate 360 million paired ends reads, each 150 base pairs (bp) in length, for each sample. |
| Sapkota et al., AACR, 2021 | Blood | DNA concentration was fluorometrically measured using the Quant-it dsDNA Assay Kit (Life Technologies cat#Q33130) and DNA integrity verified visually by agarose gel electrophoresis (E-gel, Life Technologies, cat#G8008-01). Unique samples were barcoded and WGS was performed at the HudsonAlpha Institute for Biotechnology Genomic Services Laboratory using the Illumina HiSeq X10 sequencers to generate 360 million paired ends reads, each 150 base pairs (bp) in length, for each sample. Paired-end reads were aligned to the GRCh38 human reference using the Burrows-Wheeler Aligner (BWA-ALN v0.7.12) and data was cleaned following the best practice guide by the Genome Analysis Toolkit (GATK v3.4.0).  9.3 million common [minor allele frequency (MAF)≥0.05] variants; 10.2 million rare/low frequency (MAF<0.05 and minor allele count≥3) variants. |

Abbreviations: ACT, Anthracycline-induced cardiotoxocity; SNP, Single Nucleotid Polymorphism; PCR, Polymerase Chain Reaction; rt-PCR, reverse-transcriptase PCR; qrt-PCR, quantitative real-time PCR; NA, Not Available.
