# Supplementary material for: Influence of genetic biomarkers on cardiac diseases in childhood cancer survivors: a systematic review
Source: Pharmacogenomics J. 2025 May 24;25(3):15. doi: 10.1038/s41397-025-00369-y (PMC12103300; doi:10.1038/s41397-025-00369-y)
Supplement: Supplementary file 3 — Supplementary Table 3 [file 41397_2025_369_MOESM3_ESM.docx]

**Supplementary Table 3.** Outcome definitions of the 20 included studies

| Study | Study design | Definition |
| --- | --- | --- |
| Sági et al., BMC Cancer, 2018 | Case-control | LVEF and LVFS data were analyzed in follow-up categories: 1) at the diagnosis (used as a control); 2) in acute phase: during the intensive chemotherapy phase; 3) during oral maintenance chemotherapy; 4) at the end of the treatment, which is after the oral maintenance chemotherapy period completed 2 or 3 years after the diagnosis; 5) from the end of the treatment until 5 years after the diagnosis; 6) 510 years after the diagnosis; 7) 10–15 years after the diagnosis; 8) more than 15 years after the diagnosis;  The worst heart function of each patient was used to define patients for the case-control type study.  The alteration of LVFS was computed and analyzed as dichotomous variable, which was defined as the difference between the LVFS value at diagnosis and at the end of the treatment. In this study, patients with decreased LVFS were compared to those with increased LVFS.  Cases definition: survivors with a LVFS≤ 28% at any time point during the follow-up.  Controls definition: survivors who received the same chemotherapy but never had a LVFS≤ 28%. |
| Blanco et al., JCO, 2012 | Matched case-control | Cardiomyopathy definition: cardiac compromise by presenting with symptoms (e.g., dyspnea, orthopnea, fatigue) and/or signs (e.g., edema, hepatomegaly, rales) of cardiac decompensation or, in the absence of symptoms/signs, if they had LVEF≤ 40% and/or LVFS≤ 28%.  Cases definition: CCS who developed cardiomyopathy after completion of cancer therapy and were alive at study participation.  Controls definition: CCS without cardiomyopathy, who had no symptoms or signs of cardiac compromise and had normal echocardiographic features or no clinical indication for echocardiographic examination because of lack of exposure to anthracyclines or chest radiation. |
| Visscher et al., Pediatr Blood Cancer, 2013 | Case-control | ACT definition: early- or late-onset LV dysfunction measured by echocardiogram (LVFS) and/or symptoms requiring intervention based on CTCAE v3.  Cases definition: CCS with a LVFS ≤ 26% at any time during or after anthracycline therapy.  Controls definition: CCS with a LVFS ≥ 30% during and after therapy, with follow-up of more than 5 years after completion of anthracycline therapy.  To exclude transient acute cardiotoxicity, only echocardiograms obtained 21 days or more after a dose were used. |
| Singh et al., Cancer, 2020 | Matched case-control | Cardiomyopathy definition: cardiac compromise by presenting with symptoms (e.g., dyspnea, orthopnea, fatigue) and/or signs (e.g., edema, hepatomegaly, rales) of cardiac.  Cases definition: anthracycline-exposed CCS who developed cardiomyopathy.  Controls definition: anthracycline-exposed CCS with no signs or symptoms of cardiomyopathy. |
| Blanco et al., Cancer. 2008 | Nested matched case-control | CHF ascertained through self-report by the study participants, validated by a telephone interview, during which the signs and symptoms of CHF as well as the use of medications for management of the CHF were reviewed. The responses from the script were reviewed independently by 2 physicians, and the information was used to validate CHF.  Cases: CCS who survived at least 5 years after cancer diagnosis presenting CHF.  Controls: CCS who survived at least 5 years after cancer diagnosis and did not present signs of CHF. |
| Hildebrandt et al., Nature, 2017 | Matched case-control | Cardiotoxicity not defined.  Cases definition: CCS either with a LVEF 45–50% and presenting symptoms and other echocardiogram findings considered by a cardiologist to warrant cardiac medications; or with a LVEF≤ 45% and/or LVFS≤ 25% on at least two echocardiograms. All patients included because they were started on cardiac medications had chart review to assure that medications were started for cardiac dysfunction and not hypertension without echocardiogram abnormalities. Patients who had a single low LVEF or LVSF that returned to LVEF > 55% without the use of cardiac medications were not included as cases.  Controls definitions: CCS with LVEF >55% and LVSF> 28% with at least two echocardiograms obtained more than 5 years off treatment. A patient with a single discrepant low LVEF or LVFS with subsequent normal echocardiograms not on medications were considered a control. |
| Aminkeng et al., Nat Genet. 2015 | Case-control | ACT definition: early- or late-onset LV dysfunction measured by echocardiogram (LVFS) and/or symptoms (dyspnea, orthopnea and/or fatigue) and/or signs (edema, hepatomegaly and/or rales) requiring intervention based on CTCAE v3.  Cases definition: CCS with a grade ≥ 2 ACT, LVFS ≤ 24% for asymptomatic ACT.  Controls definition: CCS with no signs or symptoms of cardiac compromise at study participation (grade 0) and LVFS≥ 30% with ≥5 years of follow-up after the end of anthracycline treatment.  To exclude transient acute cardiotoxicity, only echocardiograms obtained 21 days or more after a dose were used. |
| Visscher et al., Pharmacogenomics, 2015 | Case-control | ACT definition: early- or late-onset LV dysfunction measured by echocardiogram (LVFS) and/or symptoms requiring intervention based on CTCAE v3.  Cases definition: CCS with a LVFS ≤ 26% at any time during or after anthracycline therapy.  Controls definition: CCS with a LVFS ≥ 30% during and after therapy, with follow-up of more than 5 years after completion of anthracycline therapy.  To exclude transient acute cardiotoxicity, only echocardiograms obtained 21 days or more after a dose were used. |
| Wang et al., JCO, 2016 | Matched case-control | Cardiomyopathy definition: cardiac compromise by presenting with symptoms (e.g., dyspnea, orthopnea, fatigue) and/or signs (e.g., edema, hepatomegaly, rales) of cardiac decompensation or, in the absence of symptoms/signs, if they had LVEF≤ 40% and/or LVFS≤ 28%.  Cases definition: CCS who developed cardiomyopathy after completion of cancer therapy and were alive at study participation.  Controls definition: CCS without cardiomyopathy. |
| Wang et al., JCO, 2014 | Matched case-control | Cardiomyopathy definition: cardiac compromise by presenting with symptoms (e.g., dyspnea, orthopnea, fatigue) and/or signs (e.g., edema, hepatomegaly, rales) of cardiac decompensation or, in the absence of symptoms/signs, if they had LVEF≤ 40% and/or LVFS≤ 28%.  Cases definition: CCS who developed cardiomyopathy after completion of cancer therapy and were alive at study participation.  Controls definition: CCS without cardiomyopathy. |
| Wang et al., JCO, 2022 | Case-control | Cardiomyopathy, CHF, heart transplantation, and medications were classified and graded using the CTCAE.  Only those outcomes graded as severe (grade 3; self-reported cardiomyopathy or CHF, plus medications), life-threatening (grade 4; requiring heart transplantation), or fatal (grade 5) were included. |
| Visscher et al., Journal of Clinical Oncology, 2012 | Case-control | ACT definition: early- or late-onset LV dysfunction measured by echocardiogram (LVFS) and/or symptoms requiring intervention based on CTCAE v3.20.  Cases definition: CCS with a LVFS ≤ 26% at any time during or after anthracycline therapy.  Controls definition: CCS with a LVFS ≥ 30% during and after therapy, with follow-up of more than 5 years after completion of anthracycline therapy.  To exclude transient acute cardiotoxicity, only echocardiograms obtained 21 days or more after a dose were used. |
| Chaix et al., JACC : Cardiooncology, 2020. | Nested case-control | Cardiotoxicity definition: LVEF ≤ 50% or >10% LVEF decline to ≤55% from a previous echocardiogram during follow-up.  Cases definition: patients who received low cumulative anthracycline dose (≤250 mg/m^2^) but developed either: 1) clinically defined cardiotoxicity; or 2) low LVEF of ≤55% based on American Society of Echocardiography guidelines.  Controls definition: patients with preserved cardiac function (LVEF of >55%) despite high dose anthracycline (>250 mg/m^2^). |
| Sharafeldin et al., JACC: Cardiooncology, 2023 | Matched case-control | Cardiomyopathy definition: cardiac compromise presenting with signs and/or symptoms (dyspnea, orthopnea, fatigue, edema, hepatomegaly, and/or rales) or, in the absence of signs or symptoms, with echocardiographic features of left ventricular dysfunction (LVEF≤ 40% and/or LVFS ≤28%).  Severe cardiomyopathy: symptomatic cardiac dysfunction with an LVEF ≤40% or LVFS≤ 25%.  Mild cardiomyopathy: all other cases |
| Krajinovic et al., The Pharmacogenomics Journal, 2016 | Cohort | Only LVFS and LVEF were analyzed, as these two parameters were significantly associated with genotypes in the ALL group. LV volumes were used for LVEF calculation and were estimated from diastolic and systolic dimensions using Teichholz’ formula. Measurements obtained at ≥3 years after diagnosis were taken for genotype–phenotype comparison corresponding to the time between diagnosis and echocardiography of the ALL cohort. |
| Semsei A., Cell Biol. Int, 2012 | Cohort | Patients were followed-up by echocardiography to assess LV function by measuring left ventricular end-diastolic-dimension and left ventricular end-systolic dimension. LVFS was calculated from these 2 data.  LVFS was analyzed at 3 time-points, first, at the time of diagnosis, secondly at the end of the treatment, that is, at a median of 2 years after diagnosis, while the third data-point was determined at the time of the latest follow-up. |
| Lipshultz S., Cancer, 2013 | Cohort | Echocardiograms were obtained at diagnosis, after doxorubicin therapy, and every 2 years thereafter at local treatment sites and centrally re-measured at a single facility by study staff blinded to treatment status.  Left ventricular status was assessed with LV end systolic and end-diastolic dimensions; LV mass; LV end-systolic and end-diastolic posterior wall thicknesses; LV thickness-to-dimension ratio; and LV fractional shortening, an index of LV systolic performance influenced by heart rate, LV preload, LV afterload, and LV contractility.  Echocardiographic measurements were standardized with Z-scores. Z-scores were calculated from the difference between LV outcome values in patients and known values in healthy children, divided by the standard deviation of a distribution of values in healthy children. |
| Petrykey et al., Pharmacogenomics, 2021 | Cohort | Echocardiographic parameters for the quantitation of LV morphology and function included the LV end-diastolic diameter, LVFS) and LVEF. Two measurements of the LVEF were studied – LVEF M-mode and LVEF 2D (Simpson). |
| Sapkota et al., JNCI J Natl Cancer Inst, 2022 | Cohort and case-control | LVEF as a quantitative trait, according to the American Society of Echocardiography guidelines.  When multiple EF measurements were available, we used the lowest LVEF of each survivor to reflect the most severe form of CCD known for the survivor.  In SJLIFE patients, CCD was clinically assessed based on LVEF and pharmacologic treatment. Using modifications of the National CTCAE v4.03, CCD was classified as moderate (grade 2: resting LVEF < 50%-40% or 10%-19% absolute drop from baseline), severe or disabling (grade 3: resting LVEF < 39%-20%, or >20% absolute drop from baseline, or medication initiated), life-threatening (grade 4: resting LVEF < 20%; refractory or poorly controlled heart failure; intervention such as ventricular assist device, intravenous vasopressor support; or heart transplant indicated), or fatal (grade 5: death);  In CCSS, participants completed a multi-item questionnaire at baseline and follow-up surveys. Using the CTCAE, CHF was graded as moderate (grade 2: self-reported CHF not requiring medication), severe or disabling (grade 3: cardiomyopathy of CHF requiring medication), life-threatening (grade 4: cardiac transplantation), or fatal (grade 5: death as a result of heart failure). |
| Sapkota et al., AACR, 2021 | Cohort | LVEF as a quantitative trait, according to the American Society of Echocardiography guidelines.  cardiomyopathy was clinically assessed and graded using a modified version of the National Cancer Institute’s CTCAE v4.03, and classified as mild (grade 1), moderate (grade 2), severe or disabling (grade 3), life- threatening (grade 4), or fatal (grade 5). |

Abbreviations: ACT, Anthracyclines-Induced Cardiotoxicity; LV: Left-ventricle; LVFS, Left-Ventricular Fractional Shortening; LVEF, Left-ventricular Ejection Fraction; CTCAE, Common Terminology Criteria for Adverse Events; CHF, Congestive Heart Failure; CCD, Cancer treatment–induced cardiac dysfunction; NA, Not Available.
