# Supplementary material for: Influence of genetic biomarkers on cardiac diseases in childhood cancer survivors: a systematic review
Source: Pharmacogenomics J. 2025 May 24;25(3):15. doi: 10.1038/s41397-025-00369-y (PMC12103300; doi:10.1038/s41397-025-00369-y)
Supplement: Supplementary file 4 — Supplementary Table 4 [file 41397_2025_369_MOESM4_ESM.docx]

**Supplementary Table 4.** Statistical methods and selection procedure of the 20 included studies

| **Study** | **Sequencing method** | **Statistical Analysis** | **Variables for Matching / adjustment** | **Selection procedure** | **Correction for multiplicity** | **Performance evaluation** | **Validation of the results** |
| --- | --- | --- | --- | --- | --- | --- | --- |
| **Sági et al., BMC Cancer, 2018** | Candidate gene | Logistic regression for case-control analysis | Adjustment: age at the time of diagnosis, gender, chemotherapy protocols, risk groups, and cumulative dose of anthracycline | NA | Benjamini-Hochberg FDR, p≤ 8.90 x 10^-3^ | NA | NA |
|  |  | Linear regression for follow-up analysis |  |  |  |  |  |
| **Blanco et al., JCO, 2012** | Candidate gene | Conditional logistic regression | Matching: cancer diagnosis, year of diagnosis (± 5 years), race/ethnicity, and duration of follow-up for controls to exceed time from cancer diagnosis to cardiomyopathy for index patient case  Adjustment: cumulative ANT dose, sex, age at cancer | NA | Bonferroni correction for multiple comparisons (p< 0.025) when analyzing the association with the two SNPs. | NA | NA |
| **Visscher et al., Pediatr Blood Cancer, 2013** | Candidate Gene | Single SNP Logistic regression | Adjustment: Cumulative anthracycline dose, age at start of treatment, gender and radiation therapy to the heart included as covariates | NA | NA | ROC curve and -statistic (area under the curve, AUC) calculation | Tested in the replication cohort (Dutch-EKZ and Canadian-CPNDS patients combined) |
|  |  | Logistic regression | including multiple genetic variants and/or clinical variables were trained in the original cohort | Stepwise regression with forward selection: association with ACT at p< 0.01 were retained in the final model |  |  |  |
| **Singh et al., Cancer, 2020** | Candidate gene | Conditional logistic regression | Matching: cancer diagnosis, year of diagnosis (± 5 years), race/ethnicity, and duration of follow-up for controls to exceed time from cancer diagnosis to cardiomyopathy for index patient case  Adjustment: anthracycline dose, chest radiation, age at diagnosis of primary cancer, and sex | NA | NA | NA | Gene expression analyses on cardiomyocytes and peripheral blood |
| **Blanco et al., Cancer, 2008** | Candidate gene | Conditional logistic regression | Matching: age at diagnosis (± 5 years), race/ethnicity, anthracycline dose, radiation to the heart (matched for 81% of controls; this factor was included in all models to adjust for incomplete matching) and duration of follow-up for controls to exceed time from cancer diagnosis to cardiomyopathy for index patient case  Adjustment: sex, smoking history, first recurrence of original disease, heart in radiation beam, and family history of heart disease | NA | NA | NA | NA |
| **Hildebrandt et al., Nature, 2017** | Candidate gene | Logistic regression | Matching; age at diagnosis, gender, anthracycline dose, chest radiation, and cancer type  Adjustment: follow-up time, age at diagnosis, gender, race, hypertension, anthracycline dose, chest radiation, and cancer site | Gene associated with cardiotoxicity at p≤ 0.05 | NA | NA | Expression of the identified genes was analyzed in cardiomyocytes |
| **Aminkeng et al., Nat Genet., 2015** | GWAS | Logistic regression | Adjustment: age at the start of treatment, cumulative anthracycline dose, tumor type and cardiac radiation therapy | Association with ACT: p< 1 × 10^−5^ for the discovery cohort and p< 0.006 on the replication cohort, and p< 0.05 in the independent non-European population | Multiple testing correction p< 1 × 10^−5^ for the discovery cohort.  Bonferroni correction p< 0.006 for the replication cohort | NA | Markers that reached significance threshold in the discovery cohort were tested for replication (European Dutch patients) and the SNPs significant after Bonferroni correction in the replication cohort were tested in non-European population, and in combined analysis of all patients with European ancestry in discovery and replication cohorts |
| **Visscher et al., Pharmacogenomics, 2015** | GWAS | Logistic regression | Adjustment: cumulative anthracycline dose, age at start of treatment, gender, radiation therapy, and the first two principal components, and SLC28A3 (rs7853758) and UGT1A6 (rs17863783) | Tiered analysis to identify SNPs associated with ACT at p< 0.01 in the discovery cohort that remained associated in the replication cohort at p< 0.05  For SNPs not identified in the tiered analysis, p< 0.005 in the combined cohort was considered suggestive evidence | Bonferroni corrected  p< 1.7 × 10^-5^ | ROC curve and -statistic (area under the curve, AUC) calculation | Tested in the replication cohort (Dutch-EKZ and Canadian-CPNDS patients combined) |
|  |  | Logistic regression | including multiple genetic variants and/or clinical variables were trained in the original cohort |  |  |  |  |
| **Wang et al., JCO., 2016** | GWAS | Conditional logistic regression | Matching: cancer diagnosis, year of diagnosis (± 5 years), race/ethnicity, and duration of follow-up for controls to exceed time from cancer diagnosis to cardiomyopathy for index patient case  Adjustment: cumulative ANT dose, RT exposition, sex, and age at cancer (step 1), and SNP-anthracycline interactions (Step 2) | Association with cardiomyopathy: p< 0.004 | Multiple testing correction: p< 7.77 x 10^-5^ | NA | Replication on case-only design, to verify significant SNP-anthracycline interactions identified in the discovery stage  Gene expression analysis on heart samples |
| **Wang et al., JCO, 2014** | GWAS | Conditional logistic regression | Matching: cancer diagnosis, year of diagnosis (± 5 years), race/ethnicity, and duration of follow-up for controls to exceed time from cancer diagnosis to cardiomyopathy for index patient case  Adjustment: cumulative ANT dose, total RT dose, sex, age at cancer and SNP-anthracycline interactions | Association with cardiomyopathy: p< 5 x 10^-6^ | Multiple testing correction p< 5 x 10^-6^ | NA | Replication on case-only design, to verify significant SNP-anthracycline interactions identified in the discovery stage  Gene expression analysis on heart samples |
| **Wang et al., JCO, 2022** | GWAS | Cox regression | Adjustment: 10 genotype-based principal components, age at diagnosis of primary cancer, sex, chest radiation, anthracycline dose, and presence of cardiovascular risk factors (and SNP-anthracycline interactions for step 2) | Gene associated with cardiotoxicity at p≤ 5 x 10^-8^ | NA | NA | Replication on matched case-control design to verify significant SNP and SNP-anthracycline interactions identified in the discovery stage |
| **Visscher et al., JCO, 2012** | GWAS | Logistic regression | Adjustment: Cumulative dose, age, sex, and radiation therapy to the heart included as covariates as well as the first two principal components assuming an additive model | Tiered single-marker analysis to identify variants associated with ACT in the discovery cohort (p< 0.01) that remained associated in the Canadian replication cohort (p< 0.01)  Using stepwise logistic regression with forward selection including covariates, SNPs were retained with p< 0.01 in the final multi-SNP model | The overall significance threshold accounting for multiple testing was calculated at 1.5 x 10^-4^ using the simpleM correction | ROC analysis was performed for each sample from the multimarker regression model. | The top SNP was further evaluated in the Dutch-EKZ cohort and in all three cohorts combined. SNPs with p< 0.01 in the single-SNP test for the combined Canadian cohort were considered for potential inclusion in a multimarker risk prediction model |
| **Chaix et al., JACC : Cardiooncology, 2020** | WES | Burden CMC. SKAT. SKAT-O.  Logistic regression for associated genes | Adjustment: sex, cancer diagnosis, age at the start of anthracycline, use of dexrazoxane, chest radiation, duration of follow-up from first anthracycline dose, and the first 2 principal components inferring ethnicity | Gene association with cardiotoxicity p< 0.001 by at least 2 gene-collapsing methods and genes in biologically relevant pathways with an association of p< 0.001 by at least 1 method | Bonferroni correction: p< 2.87 x 10^-6^ | Random forest model on the discovery cohort.  ROC AUC, sensitivity, specificity, positive predictive value, negative predictive value, false positive rate, false negative rate and misclassification rate calculation | Replication on 1:1 propensity-matched case-control design, comprising a subset of patients with recent enrollment in the PCS2 study |
| **Sharafeldin et al., JACC: Cardiooncology, 2023** | WES | Ordinal logistic regression | Matching: cancer diagnosis, year of diagnosis (±10 years), race/ethnicity, and duration of cardiomyopathy-free follow-up equal to or greater than the case.  Adjustment: cumulative anthracycline dose, chest radiation, cardiovascular risk factors (yes/no), cancer diagnosis, age at cancer diagnosis, sex, race/ethnicity, and 3 principal components. | Identification of gene-level SNP–SNP interactions using logic regression based on a logit link and a random seed. Optimal model size was derived using 10-fold cross-validation up to a maximum size of 2 trees and 5 leaves/tree. The best solution based on a lower deviance was selected as the final model producing the gene-level SNP set.  The logic regression searches for Boolean combinations (AND, OR, and NOT) of binary indicators (i.e., SNPs) within each set (i.e., gene). The binary indicators of SNPs are obtained by creating 2 dummy variables for the heterozygous and homozygous variant genotypes. | Bonferroni correction: p< 6.93 x 10^-6^ | NA | Replication set 1: nonoverlapping cases (n = 32) and matched control subjects (n = 173), enrolled in the COG study ALTE03N1 following the enrollment of the discovery set.  Replication set 2: from the BMTSS. Cases (n = 135) were diagnosed with heart failure (HF). Control s (n = 262) were matched on (≤2 per case) on race/ethnicity, type of transplant, cancer diagnosis, year of cancer diagnosis, and duration of cardiomyopathy-free follow-up (equal to or greater than the matched case).  Replication set 3: a cohort of 5,589 non-Hispanic White participants from the CCSS. Cases (n=229) were diagnosed with grade ≥3 HF. |
| **Krajinovic et al., The Pharmacogenomics Journal, 2016** | Candidate gene | Analysis of variance or t-test | NA | Either an association sustained multiple testing correction (FDR ≤0.05) with at least one of the tested parameters, or association was preferentially seen in high-risk patients who received higher cumulative doxorubicin doses | multiple testing correction (FDR ≤0.05) | NA | Findings were validated in set of 44 ALL patients who underwent treatment with DFCI 95-01 protocol |
| **Semsei A et al., Cell Biol. Int, 2012** | Candidate gene | Linear regression | Adjustment: Gender, age at diagnosis, clinical centre, total anthracyclines dose, dexrazoxane, chemotherapy protocol. | Gene association with LVFS p< 0.005 | Bonferroni correction p< 0.005 | NA | NA |
| **Lipshultz S et al., Cancer. 2013** | Candidate gene | t-tests or Wilcoxon rank-sum tests | The Z-scores for LV mass, LV end-systolic and end diastolic posterior wall thicknesses, and LV dimensions were adjusted for body-surface area, and Z-scores for LV fractional shortening were adjusted for age at echocardiography. | 0.05 | NA | NA | NA |
| **Petrykey et al., Pharmacogenomics, 2021** | WES | Wald statistic and linear regression for common variants | Adjustment: age at the time of diagnosis, time since the end of treatment; sex; treatment protocol; and a risk-treatment scores | Common variants in genes involved in cardiac system functioning  p< 0.0006 | Benjamini–Hochberg procedure for FDR was p< 0.05  Bonferroni p-value corrected p < 0.0006 | NA | Analyses of top-ranking rare variants in ALL survivors of European ancestry enrolled in the SJLIFE cohort |
| **Sapkota et al., JNCI, 2022** | WGS | Linear regression | Adjustment: sex, age at cancer diagnosis, age at last follow-up, cumulative anthracycline dose, average heart radiation dose, and the top 20 genotype-based principal components | p< 5 x 10^-8^ | NA | NA | Variants that met the statistical significance thresholds in the discovery analysis were tested in 2 replication cohorts including 301 survivors of African ancestry from the SJLIFE (for LVEF and CCD) and an additional 4020 of European ancestry from the CCSS (for self-reported CHF) |
|  |  | Logistic regression |  | P<0.05 considered statistically significant | NA | NA |  |
|  |  | SKAT-O for rare variants | NA | p< 0.05 | Multiple test adjustments with a cutoff value of FDR <0.05 |  |  |
| **Sapkota et al., AACR, 2021** | WGS | Linear regression for common variants | Adjustment: sex, age at cancer diagnosis, age at last follow-up, cumulative anthracycline dose, average heart radiation dose and the top 10 principal components | p< 5×10^-8^ considered significant at the genome-wide level | NA | NA | Findings were replicated on 1645 patients from the SJLIFE survivors of European ancestry exposed to cardiotoxic therapies (radiotherapy to the heart and anthracyclines) |
|  |  | SKAT-O for rare and low-frequency variants | NA | p< 7.3×10^-8^ | NA |  |  |
|  |  | Logistic regression for both common, low-frequency and rare variants | Matching on PS deciles.  PS calculation: by regressing each SNP on all the sex, age at cancer diagnosis, age at last follow-up, cumulative anthracycline dose, average heart radiation dose and the top 10 principal components | NA | NA |  |  |

Abbreviations: ACT, Anthracycline-induced cardiotoxicity; SNP, Single-nucleotide polymorphism; CHF, Congestive heart failure; CCD: Cancer treatment–induced cardiac dysfunction; CMC, Combined Multivariate and Collapsing; SKAT, Sequence kernel association test; SKAT-O, sequence kernel association test-optimized; ALL, Acute lymphocytic leukemia; SJLIFE, St. Jude Lifetime; DFCI, Dana-Farber Cancer Institute; PCS2: Preventing Cardiac Sequelae in Pediatric Cancer Survivors; CCSS, Childhood Cancer survivor Study; BMTSS, Blood or Marrow Transplant Survivor Study; FDR, False discovery rate; PS, Propensity Score; NA, Not Available
