# Supplementary material for: Influence of genetic biomarkers on cardiac diseases in childhood cancer survivors: a systematic review
Source: Pharmacogenomics J. 2025 May 24;25(3):15. doi: 10.1038/s41397-025-00369-y (PMC12103300; doi:10.1038/s41397-025-00369-y)
Supplement: Supplementary file 5 — Appendix [file 41397_2025_369_MOESM5_ESM.docx]

# Appendix A: Study search and selection procedure

We performed a systematic search to identify studies highlighting associations between cardiac diseases and genetics in patients treated with anthracyclines and/or radiations for childhood cancer before 21 years old. The search was performed on 11 April 2023, then updated on 29 August 2024, using two electronic databases, NCBI PubMed and Scopus, and restricted to English. No year restriction was applied.

Keywords were constructed using Medical Subject Headings (MeSH) terms and other free-text terms. The search strategy used the PICOS (Population, Intervention, Comparison, Outcome, Study design) framework (Table A1).

**Table A.** PICOS wording search strategy

| **PICOS** | | **Wording of search strategy** |
| --- | --- | --- |
| **Population** | Childhood Cancer Survivors | #1: Cancer OR Oncology OR neoplasms OR malignant OR tumor OR carcinoma OR leukemia OR retinoblastoma OR sarcoma OR osteosarcoma OR Wilms tumor OR nephroblastoma OR neuroblastoma OR brain neoplasms OR CNS OR central nervous system OR lymphoma OR Hodgkin disease OR Hodgkin Diseases #2: Cancer survivors OR "long-term survival" OR "followed up" OR Survivor* OR childhood cancer survivors #1 AND #2 |
| **Intervention** | Radiotherapy Chemotherapy | #1: Radiotherapy OR Radiations OR "Dose Volume" #2: Chemotherapy OR Antimetabolites OR alkylating OR Topoisomerase OR "Vinca Alkaloid" OR "Tyrosine kinase" OR Anthracyclines OR Daunorubicin OR Adriamycin OR Doxorubicin OR Mitoxantrone OR Antineoplastic agent #1 OR #2 |
| **Comparator** | None |  |
| **Outcome** | Genetic factors associated with Cardiotoxicity | #1: Heart diseases OR Heart Disorders OR Cardiac Diseases OR Cardiac Disorders OR cardiovascular OR Cardiotoxicit* OR Cardiomyopathy* OR Heart failure OR arrhythmia OR Conduction disorders OR Valvular diseases OR heart valve disease OR ischemia OR myocardial infarction OR Coronary artery disease OR Ischemic heart diseases OR Angina OR Heart attack OR Pericarditis OR cardiac toxicity #2: genetics OR Genotype OR gene expression OR Transcript* OR "SNPs" OR "single nucleotid polymorphism" OR "copy number variations" OR "Sequencing" OR "rna seq" OR "GWAS" OR "genetic association studies" OR "Biomarkers" OR "genome-wide association study" #1 AND #2 |
| **Study Design** | Cohorts, case-control | Cohort Stud* OR Observational Stud* OR Case control studi* OR Prospective Stud* OR Retrospective Stud* |

The selection procedure was translated to the electronic bibliographic databases as follow:

**NCBI PubMed search strategy**

#1 (((((((((((((((((((((Cancer) OR (Oncology)) OR (neoplasms)) OR (malignant)) OR (tumor)) OR (carcinoma)) OR (leukemia)) OR (retinoblastoma)) OR (sarcoma)) OR (osteosarcoma)) OR (Wilmstumor)) OR (nephroblastoma)) OR (neuroblastoma)) OR (brain neoplasms)) OR (CNS)) OR (central nervous system)) OR (lymphoma)) OR (Hodgkin disease)) OR (Hodgkin Diseases)))) AND ((((Cancer survivors) OR ("long-term survival")) OR ("followed up")) OR (Survivor*))

#2 ((((Radiotherapy) OR (Radiations)) OR ("Dose Volume")) OR ((((((((((((Chemotherapy) OR (Antimetabolites)) OR (alkylating)) OR (Topoisomerase)) OR ("Vinca Alkaloid")) OR ("Tyrosine kinase")) OR (Anthracyclines)) OR (Daunorubicin)) OR (Adriamycin)) OR (Doxorubicin)) OR (Mitoxantrone)) OR (Antineoplastic agent)))

#3 (((((((((((((((((((((Heart diseases) OR (Heart Disorders)) OR (Cardiac Diseases )) OR (Cardiac Disorders)) OR (cardiovascular)) OR (Cardiotoxicit*)) OR (Cardiomyopath*)) OR (Heart failure)) OR (arrythmia)) OR (Conduction disorders)) OR (Valvular diseases)) OR (heart valve disease)) OR (ischemia)) OR (myocardial infarction)) OR (Coronary artery disease)) OR (Ischemic heart diseases)) OR (Angina)) OR (Heart attack)) OR (Pericarditis)) OR (Cardiac toxicity))) AND (((((((((((((( genetics) OR (Genotype)) OR (gene expression)) OR (Transcript*)) OR "SNPs") OR "single nucleotid polymorphism") OR "copy number variations") OR "Sequencing") OR "rna seq") OR "GWAS") OR "genetic association studies") OR "Biomarkers") OR "genome-wide association study"))

#4 ((((((Cohort Stud*) OR (Observational Stud*)) OR (Case control studi*)) OR (Prospective Stud*)) OR (Retrospective Stud*)))

#1 AND #2 AND #3 AND #4

**Scopus search strategy**

#1 ((TITLE-ABS-KEY (((((((((((((((((((cancer) OR (oncology)) OR ( neoplasms )) OR ( malignant )) OR ( tumor )) OR ( carcinoma )) OR ( leukemia )) OR ( retinoblastoma )) OR ( sarcoma )) OR ( osteosarcoma )) OR ( "Wilms tumor" )) OR ( nephroblastoma )) OR ( neuroblastoma )) OR ( "brain neoplasms" )) OR ( CNS )) OR ( "central nervous system" )) OR ( lymphoma )) OR ( "hodgkin disease" )))) AND ( ALL (((( "long-term survival" ) OR ("Followed up")) OR ( survivor* )))))

#2 (( ALL (( ( radiotherapy ) OR ( radiations )) OR ( "Dose Volume" ))) OR ( ALL (((((((((((( chemotherapy ) OR ( antimetabolites )) OR ( alkylating )) OR ( topoisomerase )) OR ( "Vinca Alkaloid" )) OR ( "Tyrosine kinase" )) OR ( anthracyclines )) OR ( daunorubicin )) OR ( adriamycin )) OR ( doxorubicin )) OR ( mitoxantrone )) OR ( "antineoplastic agent" ))))

#3 ((TITLE-ABS ((((((((((((((((((((("heart diseases") OR ("heart disorders")) OR ("cardiac diseases")) OR ("cardiac disorders")) OR (cardiovascular)) OR (cardiotoxicit*)) OR (cardiomyopath*)) OR ("heart failure")) OR (arrythmia)) OR ("conduction disorders")) OR ("valvular diseases")) OR ("heart valve disease")) OR (ischemia)) OR ("myocardial infarction")) OR ("coronary artery disease")) OR ("ischemic heart diseases" )) OR ( angina )) OR ("heart attack")) OR ( pericarditis )) OR ("cardiac toxicity")))) AND (TITLE-ABS (((((((((((((("genome-wide association study" )) OR ( polymorphism ) OR ( genetic )) OR ( genotype )) OR ("gene expression")) OR (transcription)) OR (transcriptome)) OR (snps)) OR ("single nucleotid polymorphism")) OR ("copy num-ber variations")) OR (sequencing)) OR ("rna seq")) OR (gwas)) OR (biomarkers))))

#4 (ALL (cohort* OR "cohort stud*" OR "observational stud*" OR "case-control stud*" OR "prospective stud*" OR "Retrospective Stud*" OR "longitudinal stud*"))

#5 (KEY (mouse, AND mice, "Animal Model", "Animal Tissue", AND animal, AND non-human)) AND (LIMIT-TO (SUBJAREA, "MEDI") OR LIMIT-TO (SUBJAREA,"BIOC") OR LIMIT-TO (SUBJAREA,"PHAR") OR LIMIT-TO (SUBJAR-EA, "COMP")) AND (LIMIT-TO (DOCTYPE,"ar") OR LIMIT-TO (DOCTYPE,"re")) AND (LIMIT-TO (LANGUAGE,"English"))

#1 AND #2 AND #3 AND #4 AND NOT #5

# Appendix B: Inclusion/ exclusion criteria

Inclusion criteria:

- The study included individuals who were diagnosed with cancer prior to the age of 21 and received treatment involving anthracyclines and/ or radiation,
- The participants were diagnosed with heart disease or demonstrated reduced heart function,
- The diagnosis of CD was made after the completion of cancer treatment,
- The genetic sequencing was performed on all the enrolled patients,
- The study aimed to analyze the associations between genetic factors and the presence of cardiac dysfunction,
- The design of the study should be observational, which could be a cohort study, cross-sectional study, or case-control study.

Exclusion criteria:

- Case reports, reviews, systematic reviews, meta-analyses, practice guidelines, conference papers, correspondences and editorial articles,
- The patients were treated for cancer during adulthood,
- The participants did not experience any cardiac event or dysfunction after treatment,
- The patients presented pre-existing heart disease or predispositions to cardiac diseases,
- The study reported either no or very few reported cardiac events,
- The study did not report genetic results or only analyzed circulating biomarkers,
- The number of patients did not exceed 100,
- The study presents results on cardiac events in acute phase (during cancer treatment ) only
- The study focused on *in vitro* or animal models,
- The full text was not available in English.

# Appendix C. Data extraction form

We developed a data extraction form for collecting 14 items for the systematic review.

**Table C.** List of items of the data extraction form

| **Reference citation of the article** | |
| --- | --- |
| Study design | - Case-control study - Cohort study |
| Sequencing method | - candidate gene, - genome-wide association studies (GWAS) - whole-exome sequencing (WES) - whole-genome sequencing (WGS) |
| Sample size (case/controls) |  |
| Country of origin and/ or ethnicity |  |
| Cancer type |  |
| Method and follow-up duration |  |
| Cancer treatment information |  |
| Cancer treatment or cancer diagnosis area |  |
| Age at cancer diagnosis |  |
| Cardiac outcome definition |  |
| Definition of cases and controls |  |
| Gene selection method |  |
| Statistical analysis method |  |
| Selected genes and variants, and risk-associated with CD |  |

# Appendix D: References of (1) included studies and (2) eligible studies but not included

**Table D1.** References of included studies (n=20)

| Study | Reference |
| --- | --- |
| Canadian Pharmacogenomics Network for Drug Safety & Dutch-EKZ cohort | Visscher H, Ross CJD, Rassekh SR, Barhdadi A, Dubé MP, Al-Saloos H, et al. Pharmacogenomic prediction of anthracycline-induced cardiotoxicity in children. J Clin Oncol. 1 mai 2012;30(13):1422‑8. |
| Canadian Pharmacogenomics Network for Drug Safety & Dutch-EKZ cohort | Visscher H, Ross CJD, Rassekh SR, Sandor GSS, Caron HN, van Dalen EC, et al. Validation of variants in SLC28A3 and UGT1A6 as genetic markers predictive of anthracycline-induced cardiotoxicity in children. Pediatr Blood Cancer. août 2013;60(8):1375‑81. |
| Canadian Pharmacogenomics Network for Drug Safety & Dutch-EKZ cohort | Visscher H, Rassekh SR, Sandor GS, Caron HN, van Dalen EC, Kremer LC, et al. Genetic variants in SLC22A17 and SLC22A7 are associated with anthracycline-induced cardiotoxicity in children. Pharmacogenomics. 2015;16(10):1065‑76. |
| Canadian patient populations | Aminkeng F, Bhavsar AP, Visscher H, Rassekh SR, Li Y, Lee JW, et al. A coding variant in RARG confers susceptibility to anthracycline-induced cardiotoxicity in childhood cancer. Nat Genet. sept 2015;47(9):1079‑84. |
| The Childhood Cancer Survivor Study (CCSS) | Blanco JG, Leisenring WM, Gonzalez-Covarrubias VM, Kawashima TI, Davies SM, Relling MV, et al. Genetic polymorphisms in the carbonyl reductase 3 geneCBR3 and the NAD(P)H:quinone oxidoreductase 1 geneNQO1 in patients who developed anthracycline-related congestive heart failure after childhood cancer. Cancer. 15 juin 2008;112(12):2789‑95, |
| The Childhood Cancer Survivor Study (CCSS) | Blanco JG, Sun CL, Landier W, Chen L, Esparza-Duran D, Leisenring W, et al. Anthracycline-Related Cardiomyopathy After Childhood Cancer: Role of Polymorphisms in Carbonyl Reductase Genes—A Report From the Children’s Oncology Group. JCO. 1 mai 2012;30(13):1415‑21. |
| COG-ALTE03N1 | Wang X, Liu W, Sun CL, Armenian SH, Hakonarson H, Hageman L, et al. Hyaluronan synthase 3 variant and anthracycline-related cardiomyopathy: a report from the children’s oncology group. J Clin Oncol. 1 mars 2014;32(7):647‑53. |
| COG-ALTE03N1; NCT00082745 | Wang X, Sun CL, Quiñones-Lombraña A, Singh P, Landier W, Hageman L, et al. CELF4 Variant and Anthracycline-Related Cardiomyopathy: A Children’s Oncology Group Genome-Wide Association Study. JCO. 10 mars 2016;34(8):863‑70. |
| ALTE03N1; Key Adverse Events After Childhood Cancer; clinicaltrials.gov identifier NCT00082745 | Singh P, Wang X, Hageman L, Chen Y, Magdy T, Landier W, et al. Association of GSTM1 null variant with anthracycline‐related cardiomyopathy after childhood cancer—A Children’s Oncology Group ALTE03N1 report. Cancer. sept 2020;126(17):4051‑8. |
| St. Jude Lifetime Cohort (SJLIFE) | Sapkota Y, Qin N, Ehrhardt MJ, Wang Z, Chen Y, Wilson CL, et al. Genetic Variants Associated with Therapy-Related Cardiomyopathy among Childhood Cancer Survivors of African Ancestry. Cancer Research. 1 mai 2021;81(9):2556‑65. |
| Preventing Cardiac Sequelae in Pediatric Cancer Survivors (PCS2) | Chaix MA, Parmar N, Kinnear C, Lafreniere-Roula M, Akinrinade O, Yao R, et al. Machine Learning Identifies Clinical and Genetic Factors Associated With Anthracycline Cardiotoxicity in Pediatric Cancer Survivors. JACC: CardioOncology. déc 2020;2(5):690‑706. |
| Hungarian pediatric oncology centers & Second Department of Pediatrics, Semmelweis University | Sági JC, Egyed B, Kelemen A, Kutszegi N, Hegyi M, Gézsi A, et al. Possible roles of genetic variations in chemotherapy related cardiotoxicity in pediatric acute lymphoblastic leukemia and osteosarcoma. BMC Cancer. déc 2018;18(1):704. |
| MD Anderson Cancer Centre b | Hildebrandt MAT, Reyes M, Wu X, Pu X, Thompson KA, Ma J, et al. Hypertension Susceptibility Loci are Associated with Anthracycline-related Cardiotoxicity in Long-term Childhood Cancer Survivors. Sci Rep. 29 août 2017;7:9698. |
| QcALL cohort (Quebec, Canada) | Krajinovic M, Elbared J, Drouin S, Bertout L, Rezgui A, Ansari M, et al. Polymorphisms of ABCC5 and NOS3 genes influence doxorubicin cardiotoxicity in survivors of childhood acute lymphoblastic leukemia. Pharmacogenomics J. nov 2016;16(6):530‑5. |
| Hungarian pediatric oncology center | Semsei AF, Erdelyi DJ, Ungvari I, Csagoly E, Hegyi MZ, Kiszel PS, et al. ABCC1 polymorphisms in anthracycline-induced cardiotoxicity in childhood acute lymphoblastic leukaemia. Cell Biol Int. 1 janv 2012;36(1):79‑86. |
| Dana-Farber Cancer Institute (DFCI) | Lipshultz SE, Lipsitz SR, Kutok JL, Miller TL, Colan SD, Neuberg DS, et al. Impact of hemochromatosis gene mutations on cardiac status in doxorubicin-treated survivors of childhood high-risk leukemia: HFE Mutations and Survivor Cardiac Status. Cancer. 1 oct 2013;119(19):3555‑62. |
| St. Jude Lifetime Cohort (SJLIFE) | Sapkota Y, Ehrhardt MJ, Qin N, Wang Z, Liu Q, Qiu W, et al. A Novel Locus on 6p21.2 for Cancer Treatment–Induced Cardiac Dysfunction Among Childhood Cancer Survivors. JNCI: Journal of the National Cancer Institute. 1 août 2022;114(8):1109‑16. |
| PETALE study | Petrykey K, Rezgui AM, Guern ML, Beaulieu P, St-Onge P, Drouin S, et al. Genetic factors in treatment-related cardiovascular complications in survivors of childhood acute lymphoblastic leukemia. Pharmacogenomics. 22(14):885‑901. |
| CCSS & COG-ALTE03N1 | Wang X, Singh P, Zhou L, Sharafeldin N, Landier W, Hageman L, et al. Genome-Wide Association Study Identifies ROBO2 as a Novel Susceptibility Gene for Anthracycline-Related Cardiomyopathy in Childhood Cancer Survivors. JCO. 12 déc 2022;JCO.22.01527. |
| COG & CCSS | Sharafeldin N, Zhou L, Singh P, Crossman DK, Wang X, Hageman L, et al. Gene-Level Analysis of Anthracycline-Induced Cardiomyopathy in Cancer Survivors. JACC CardioOncol. 14 sept 2023;5(6):807‑18. |

**Table D2.** References of eligible studies but not included (n=21)

| References | Exclusion criteria |
| --- | --- |
| Wojnowski L, Kulle B, Schirmer M, Schlüter G, Schmidt A, Rosenberger A, et al. NAD(P)H oxidase and multidrug resistance protein genetic polymorphisms are associated with doxorubicin-induced cardiotoxicity. Circulation. 13 déc 2005;112(24):3754‑62. | Adult cancer |
| Rajić V, Aplenc R, Debeljak M, Prestor VV, Karas-Kuzelicki N, Mlinaric-Rascan I, et al. Influence of the polymorphism in candidate genes on late cardiac damage in patients treated due to acute leukemia in childhood. Leuk Lymphoma. oct 2009;50(10):1693‑8 | Small number of Childhood Cancer Survivor |
| Dube S, Rajan A, Yalamanchili S, Thomas A, Abbott L, Benz P, et al. Single nucleotide polymorphisms in sarcomeric protein genes: Association with chemotherapy induced cardiomyopathy. Internet Journal of Cardiology. 2011;10(1). | Adults cancer |
| Volkan-Salanci B, Aksoy H, Kiratli PÖ, Tülümen E, Güler N, Öksüzoglu B, et al. The relationship between changes in functional cardiac parameters following anthracycline therapy and carbonyl reductase 3 and glutathione S transferase Pi polymorphisms. J Chemother. oct 2012;24(5):285‑91. | Not childhood cancer survivors |
| Cascales A, Pastor-Quirante F, Sánchez-Vega B, Luengo-Gil G, Corral J, Ortuño-Pacheco G, et al. Association of anthracycline-related cardiac histological lesions with NADPH oxidase functional polymorphisms. Oncologist. 2013;18(4):446‑53. | Autopsy |
| Schneider BP, Shen F, Gardner L, Radovich M, Li L, Miller KD, et al. Genome-Wide Association Study for Anthracycline-Induced Congestive Heart Failure. Clin Cancer Res. 1 janv 2017;23(1):43‑51. | Adult cancer |
| Skitch A, Mital S, Mertens L, Liu P, Kantor P, Grosse-Wortmann L, et al. Novel approaches to the prediction, diagnosis and treatment of cardiac late effects in survivors of childhood cancer: a multi-centre observational study. BMC Cancer. 3 août 2017;17(1):519. | No genetic association |
| Megías-Vericat JE, Montesinos P, Herrero MJ, Moscardó F, Bosó V, Rojas L, et al. Impact of NADPH oxidase functional polymorphisms in acute myeloid leukemia induction chemotherapy. Pharmacogenomics J. avr 2018;18(2):301‑7. | Adult cancer |
| Ruiz-Pinto S, Pita G, Martín M, Alonso-Gordoa T, Barnes DR, Alonso MR, et al. Exome array analysis identifies ETFB as a novel susceptibility gene for anthracycline-induced cardiotoxicity in cancer patients. Breast Cancer Res Treat. 1 janv 2018;167(1):249‑56. | Adult cancer |
| Garcia-Pavia P, Kim Y, Restrepo-Cordoba MA, Lunde IG, Wakimoto H, Smith AM, et al. Genetic Variants Associated With Cancer Therapy-Induced Cardiomyopathy. Circulation. 2 juill 2019;140(1):31‑41. | Small number of Childhood Cancer Survivor |
| Goel S, Liu J, Guo H, Barry W, Bell R, Murray B, et al. Decline in Left Ventricular Ejection Fraction Following Anthracyclines Predicts Trastuzumab Cardiotoxicity. JACC Heart Fail. sept 2019;7(9):795‑804. | Adult cancer |
| Hellmann F, Völler S, Krischke M, Jamieson D, André N, Bisogno G, et al. Genetic Polymorphisms Affecting Cardiac Biomarker Concentrations in Children with Cancer: an Analysis from the « European Paediatric Oncology Off-patents Medicines Consortium » (EPOC) Trial. Eur J Drug Metab Pharmacokinet. juin 2020;45(3):413‑22. | Cardiotoxicity in the acute phase, not after treatment completion. |
| McOwan TN, Craig LA, Tripdayonis A, Karavendzas K, Cheung MM, Porrello ER, et al. Evaluating anthracycline cardiotoxicity associated single nucleotide polymorphisms in a paediatric cohort with early onset cardiomyopathy. Cardio-Oncology. 21 mai 2020;6(1):5. | Small number of Childhood Cancer Survivor |
| Norton N, Crook JE, Wang L, Olson JE, Kachergus JM, Serie DJ, et al. AssociabeforeGenetic Variants at TRPC6 With Chemotherapy-Related Heart Failure. Front Cardiovasc Med. 2020;7:142. | Adult cancer |
| Vaitiekus D, Muckiene G, Vaitiekiene A, Sereikaite L, Inciuraite R, Insodaite R, et al. HFE Gene Variants’ Impact on Anthracycline-Based Chemotherapy-Induced Subclinical Cardiotoxicity. Cardiovasc Toxicol. janv 2021;21(1):59‑66. | Adult cancer |
| Loucks CM, Yan K, Tanoshima R, Ross CJD, Rassekh SR, Carleton BC. Pharmacogenetic testing to guide therapeutic decision-making and improve outcomes for children undergoing anthracycline-based chemotherapy. Basic Clin Pharmacol Toxicol. janv 2022;130 Suppl 1:95‑9. | Case report study |
| Sapkota Y, Liu Q, Li N, Bhatt NS, Ehrhardt MJ, Wilson CL, et al. Contribution of Genome-Wide Polygenic Score to Risk of Coronary Artery Disease in Childhood Cancer Survivors. JACC CardioOncol. juin 2022;4(2):258‑67. | No genetic association |
| Zolk O, von dem Knesebeck A, Graf N, Simon T, Hero B, Abdul-Khaliq H, et al. Cardiovascular Health Status And Genetic Risk In Survivors of Childhood Neuroblastoma and Nephroblastoma Treated With Doxorubicin: Protocol of the Pharmacogenetic Part of the LESS-Anthra Cross-Sectional Cohort Study. JMIR Res Protoc. 17 févr 2022;11(2):e27898. | Study has not yet been finalized |
| Vargas-Neri JL, Carleton B, Ross CJ, Medeiros M, Castañeda-Hernández G, Clark P. Pharmacogenomic study of anthracycline-induced cardiotoxicity in Mexican pediatric patients. Pharmacogenomics. avr 2022;23(5):291‑301. | Small number of Childhood Cancer Survivor |
| Xia P, Chen J, Sapkota Y, Scott EN, Liu Y, Hudson MM, et al. RBL2 Regulates Cardiac Sensitivity to Anthracycline Chemotherapy. JACC CardioOncol. 28 mars 2023;5(3):360‑73. | Animal model |
| Singh P, Zhou L, Shah DA, Cejas RB, Crossman DK, Jouni M, et al. Identification of novel hypermethylated or hypomethylated CpG sites and genes associated with anthracycline-induced cardiomyopathy. Sci Rep. 4 août 2023;13:12683. | DNA methylation analysis |

# Appendix E: Quality evaluation

The evaluation of the quality of the 20 included studies was performed by NA, using the Q-Genie Tool for Quality Assessment of Genetic Association Studies*. Each characteristic is rated from "Poor" (score=1) to "Excellent" (score=7). Scores ≤35 on the Q-Genie tool indicate poor quality studies, >35 and ≤45 indicate studies of moderate quality, and >45 indicate good quality studies.

**Table E.** Q-Genie criteria for study quality control

| Author, year | Rationale for study | Outcome of interest | Comparison Group | Technical classification of exposure | Non-technical classification of exposure | Other sources of bias | Sample size and power | A priori planning of analyses | Statistical methods and control for confounding | Testing of assumptions and inferences for genetic analyses | Appropriateness of inferences drawn from results | Score |
| --- | --- | --- | --- | --- | --- | --- | --- | --- | --- | --- | --- | --- |
| Sàgi et al., 2018 | 6 | 6 | 5 | 4 | 0 | 5 | 6 | 5 | 5 | 1 | 5 | 48 |
| Blanco et al., 2012 | 4 | 7 | 6 | 3 | 0 | 5 | 4 | 7 | 6 | 2 | 6 | 50 |
| Visscher et al., 2013 | 5 | 7 | 5 | 5 | 0 | 4 | 5 | 6 | 6 | 4 | 5 | 52 |
| Singh et al., 2020 | 6 | 5 | 5 | 2 | 0 | 5 | 3 | 4 | 6 | 4 | 4 | 44 |
| Blanco et al., 2008 | 4 | 7 | 5 | 5 | 0 | 5 | 3 | 3 | 4 | 5 | 5 | 46 |
| Hildebrandt et al., 2017 | 4 | 4 | 5 | 3 | 0 | 5 | 3 | 5 | 3 | 2 | 5 | 39 |
| Aminkeng et al., 2015 | 5 | 5 | 6 | 2 | 0 | 5 | 6 | 6 | 5 | 4 | 5 | 49 |
| Visscher et al., 2015 | 6 | 6 | 5 | 5 | 0 | 5 | 4 | 6 | 6 | 4 | 6 | 53 |
| Wang et al., 2016 | 6 | 5 | 6 | 5 | 0 | 5 | 6 | 6 | 5 | 5 | 6 | 55 |
| Wang et al., 2014 | 5 | 5 | 6 | 4 | 0 | 4 | 4 | 4 | 5 | 4 | 6 | 47 |
| Wang et al., 2022 | 6 | 6 | 5 | 4 | 0 | 5 | 5 | 6 | 6 | 6 | 5 | 54 |
| Visscher et al., 2012 | 5 | 7 | 6 | 5 | 0 | 4 | 4 | 5 | 6 | 6 | 5 | 53 |
| Chaix et al., 2020 | 5 | 6 | 5 | 5 | 0 | 5 | 4 | 6 | 5 | 4 | 6 | 51 |
| Sharafeldin et al., 2023 | 6 | 6 | 6 | 6 | 0 | 5 | 4 | 5 | 5 | 5 | 6 | 54 |
| Krajinovic et al., 2016 | 6 | 5 | 2 | 1 | 0 | 5 | 4 | 5 | 5 | 3 | 5 | 41 |
| Semsei et al., 2012 | 5 | 7 | 2 | 3 | 0 | 4 | 4 | 3 | 5 | 3 | 4 | 40 |
| Lipshultz et al.,2013 | 6 | 5 | 3 | 5 | 0 | 4 | 4 | 5 | 5 | 3 | 6 | 46 |
| Petrykey et al., 2022 | 7 | 6 | 4 | 4 | 0 | 5 | 4 | 5 | 5 | 4 | 6 | 50 |
| Sapkota et al., 2022 | 7 | 5 | 5 | 5 | 0 | 6 | 5 | 5 | 6 | 7 | 6 | 57 |
| Sapkota et al., 2021 | 6 | 6 | 4 | 5 | 0 | 4 | 4 | 6 | 5 | 4 | 5 | 49 |

*Sohani ZN, et al., Assessing the quality of published genetic association studies in meta-analyses: the quality of genetic studies (Q-Genie) tool. BMC Genet. dec 2015;16(1):50.
